# Macrophage interferon regulatory factor 7 aggravates the progression of metabolic dysfunction-associated steatohepatitis

**DOI:** 10.1186/s43556-026-00601-6

**Published:** 2026-10-19

**Authors:** Xuhao Ni, Qingfa Bu, Yuan Liang, Bozhou Pan, Jian Wang, Qingxiang Xu, Lei Liu, Qi Wang

**Affiliations:** 1 Department of General Surgery, First Affiliated Hospital of Anhui Medical University, Hefei, China; 2 Hepatobiliary Center, The First Affiliated Hospital of Nanjing Medical University, Nanjing, China; 3 School of Biological Science & Medical Engineering, Southeast University, Nanjing, China

**Keywords:** Metabolic dysfunction-associated steatohepatitis, Macrophages, Inflammation, IRF7

## Abstract

Metabolic dysfunction-associated steatohepatitis (MASH) represents a growing clinical burden with increasing prevalence and lacking approved pharmacological therapies. Interferon regulatory factor 7 (IRF7) is a central mediator of type I interferon-dependent immune responses, yet the precise mechanisms by which macrophage-expressed IRF7 shapes inflammatory and metabolic events during MASH pathogenesis remain incompletely defined. This study aimed to dissect the functional role of IRF7 in MASH development. We assessed IRF7 expression in human and mouse liver specimens, and utilized myeloid-specific *Irf7*-deficient mice fed with high-fat high-cholesterol diet (HFHCD) or methionine-choline-deficient diet (MCD) to recapitulate MASH pathology. IRF7 was significantly elevated in MASH-affected livers, and myeloid-specific *Irf7* deletion markedly mitigated MASH progression. Mechanistically, loss of IRF7 blunted IFN-α and IFN-β production, suppressing macrophage-derived type I interferon-dependent immune cascades. IRF7 directly bound to the promoters of pro-inflammatory *Tnfa* and *Il1b* to boost their transcription, and interacted with SMAD3 to activate *Tgfb1* transcription. The E3 ubiquitin ligase HUWE1 mediated IRF7 monoubiquitination to enhance its nuclear translocation, driving transcription of pro-inflammatory and pro-fibrotic genes. This molecular cascade aggravated hepatic steatosis and triggered hepatic stellate cell (HSC) activation, accelerating MASH deterioration. Pharmacological IRF7 inhibition effectively counteracted these pathological alterations. Collectively, myeloid IRF7 fuels MASH progression by amplifying macrophage-mediated liver inflammation, suggesting IRF7 as a promising therapeutic target against MASH.

**Supplementary Information:**

The online version contains supplementary material available at https://doi.org/10.1186/s43556-026-00601-6.

## Introduction

Metabolic dysfunction-associated steatotic liver disease (MASLD) has become the most prevalent chronic liver disease globally [1]. Metabolic dysfunction-associated steatohepatitis (MASH), which is defined by the presence of hepatic steatosis, lobular inflammation, and hepatocyte ballooning, may or may not be accompanied by perisinusoidal fibrosis [2], is associated with progressive liver injury, leading to cirrhosis and hepatocellular carcinoma [3]. Most MASLD cases are asymptomatic, heterogeneous and challenging to diagnose, even at advanced stages [4]. Existing strategies, including long-term weight loss and physical activity, are challenging to sustain, and only a few approved pharmacological treatments are available [5, 6]. Therefore, it is crucial to uncover the molecular mechanisms driving MASH progression to aid in the development of effective treatment options.

Macrophages play a critical role in MASLD progression by producing proinflammatory cytokines like TNF-α and IL-6, ect [7]. During MASH, inflammatory signals recruit circulating monocytes to the liver, where they mature into monocyte-derived macrophages, causing a marked expansion of the liver macrophage population [8]. Our previous studies revealed that type I interferon (IFN) signaling in macrophages plays a key regulatory role in MASH progression [9, 10]. Activation of type I and II interferon receptors (IFNAR and IFNGR) upon ligand binding triggers signaling cascades involving Janus family protein tyrosine kinases (JAK1, TYK2), resulting in the site-specific tyrosine phosphorylation of STAT1 and STAT2 [11–13]. STATs, in conjunction with IRF-9, assemble into a heterotrimeric transcription factor complex known as ISGF3, that can perform nucleus translocation and combine the IFN-stimulated response element (ISRE) to initiate the activation of target genes [12, 14]. To date, the specific mechanisms through which type I IFN signaling regulates macrophage immune responses during MASH remain largely unelucidated.

Interferon regulatory factors (IRFs) are critical transcriptional regulators of IFN signaling and innate or adaptive immune responses modulated by macrophages [11]. Among IRF1 ~ 9, IRF7 is a key regulator of type I IFN signaling. Phosphorylated IRF7 translocates into the nucleus binding to ISREs in target genes, promoting cytokine production, including IFN-α, IL-6 and TNF-α [15–18]. It has been reported that IRF7 plays a crucial role in heart fibrosis [19]. IRF7 has also been found to interact with SMAD3, a central mediator of TGF-β signaling related to systemic sclerosis [20]. Furthermore, Research has shown that single nucleotide polymorphisms of IRF7 are linked to systemic sclerosis and HCV infection in patients with cirrhosis [21, 22]. Another study demonstrated that the deficiency of IRF7 inhibits insulin resistance and obesity [23]. However, the role and specific mechanisms by which IRF7 in macrophages regulates MASH progression remain unknown.

Herein, our study investigated the role and mechanisms by which macrophage IRF7 regulates MASH progression using two different murine MASH models, which revealed that IRF7 expression is upregulated in hepatic macrophages of HFHCD- or MCD-fed mice. Of note, correlations between gene expression and histological scores can’t distinguish gene-specific effects from broad hepatic inflammatory activation. We prioritized IRF7 for functional interrogation because it is a central upstream regulator of type-I interferon signalling, motivating us further genetic investigation of its non-redundant role in MASH pathogenesis. Macrophage-specific *Irf7* deletion protected mice from HFHCD- and MCD-induced MASH. *Irf7* knockout suppressed macrophage type I IFN-dependent immune responses. Moreover, IRF7 directly bound the promoters of *Tnfa* and *Il1b* to drive pro-inflammatory transcription, and interacted with SMAD3 to transactivate *Tgfb1*. Then, the pro-inflammatory and profibrotic cytokine milieu promoted hepatic stellate cell activation and hepatic steatosis. The E3 ligase HUWE1 monoubiquitinated IRF7, facilitating its nuclear translocation and downstream transcription. Pharmacological IRF7 inhibition blocked MASH progression, identifying IRF7 as a promising therapeutic target for MASH.

## Results

### IRF7 expression is upregulated and IRF7 is activated in the livers of patients and mice with MASH

**Fig. 1:**
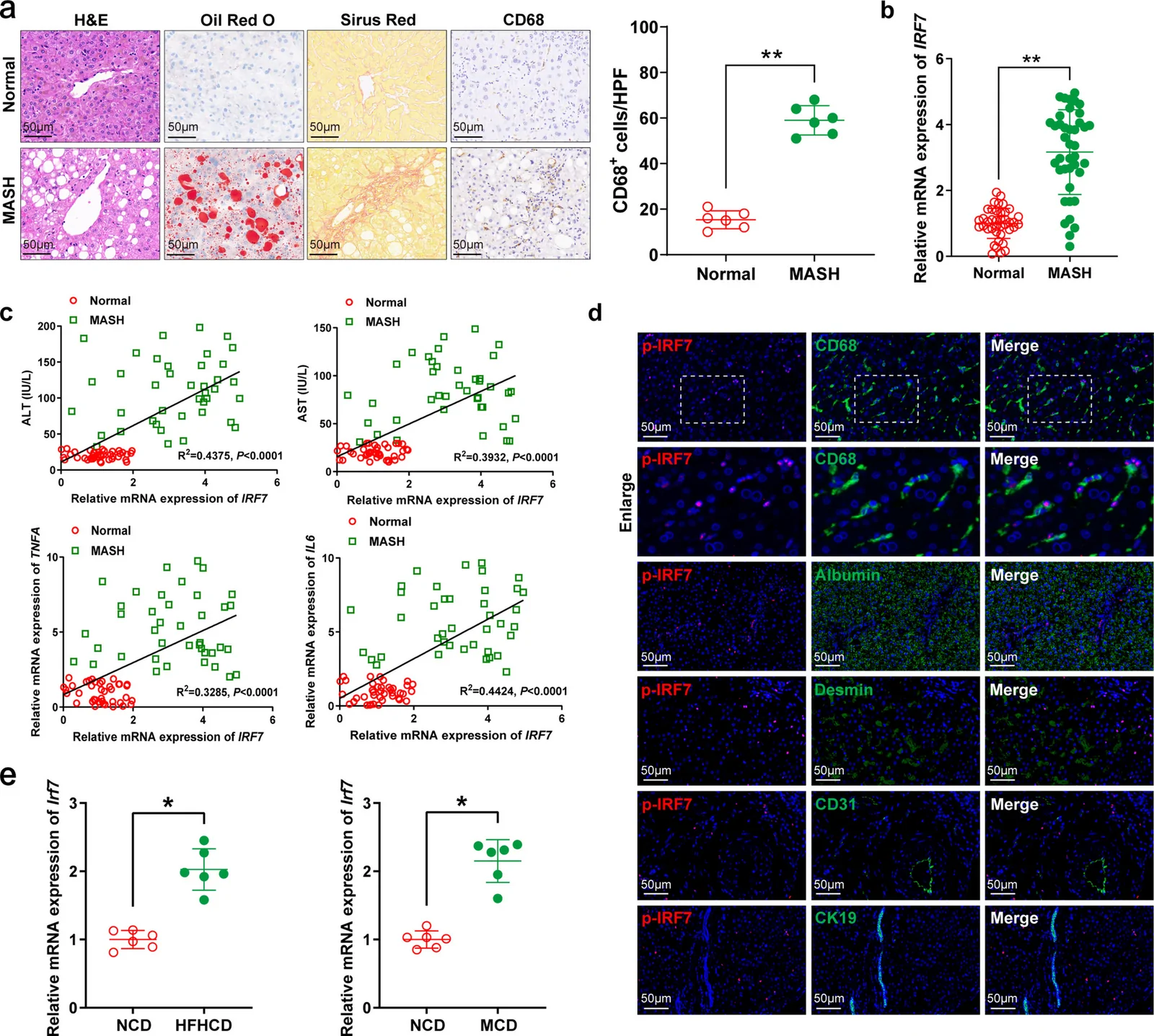
IRF7 expression is upregulated and IRF7 is activated in the livers of patients and mice with MASH. **a** Liver sections, either normal or exhibiting MASH, obtained from patients were analyzed using H&E and Oil Red O staining (*n* = 6/group; scale bar = 50 µm). **b** qRT-PCR was employed to evaluate *IRF7* gene expression in human liver samples. **c** Correlations between *IRF7* mRNA expression levels and serum ALT, AST concentrations, as well as hepatic *TNFA* and *IL6* expression, were illustrated through graphical analysis. **d** Immunofluorescence staining demonstrated the colocalization of p-IRF7 (red) with CD68 (green), Albumin (green), Desmin (green), CD31 (green), and CK19 (green) in human liver tissues. Scale bar = 50 µm. **e** The expression levels of *IRF7* in mice liver tissues subjected to HFHCD- or MCD-induced MASH were quantified via qRT-PCR; *n* = 6 mice/group. Data are presented as means ± SDs, and statistical significance was determined using Student’s t-test or ANOVA; *P < 0.05; **P < 0.01

While IRF7 deficiency has been reported to prevent diet-induced obesity and insulin resistance [20], its specific role in modulating macrophage activation during MASH remains inadequately understood. We compared IRF7 expression in liver tissues between 40 MASH patients and 40 normal patients (Table S1). Elevated macrophage quantity and gene expression of *IRF7* was detected in liver tissues from patients with MASH (Fig. 1a, b). Additionally, hepatic *IRF7* expression showed a positive correlation with serum transaminase levels (ALT and AST) and inflammatory markers (*Tnfa* and *Il6*) (Fig. 1c). Notably, dual immunofluorescence staining of liver tissues from MASH patients revealed prominent colocalization of activated IRF7 with the macrophage marker CD68. In contrast, no significant overlap was observed between activated IRF7 and markers of other cell types, such as Albumin (hepatocytes), Desmin (hepatic stellate cells), CD31 (liver sinusoidal endothelial cells), and CK19 (cholangiocytes). Further analysis confirmed that in MASH liver tissues, activated IRF7 was specifically expressed in macrophages and predominantly localized to their nuclei (Fig. 1d). Subsequent animal experiments revealed increased gene expression of *Irf7* in macrophages extracted from the livers of MASH model mice (Fig. 1e). Furthermore, to explore the clinical relevance of the above findings, patients with NASH were divided into 2 groups, an IRF7 high expression group (20/40, *IRF7*^*High*^) and an IRF7 low expression group (20/40, *IRF7*^*Low*^), according to the median value of IRF7 detected by qPCR. The degree of hepatic steatosis and fibrosis was significantly lower in the *IRF7*^*Low*^ group than the *IRF7*^*High*^ group (Fig. S1a). Moreover, Pearson correlation analysis revealed that NAFLD activity score and Fibrosis score were positively correlated with IRF7 mRNA level (Fig. S1b). Collectively, these findings highlight the upregulation and activation of IRF7 in macrophages within the liver tissues of MASH patients and the severity of liver disease in patients with MASH was positively correlated with IRF7 expression, suggesting that IRF7 may serve as a novel biomarker for MASH.

### Macrophage-specific *Irf7* deficiency attenuates MASH in mice

**Fig. 2:**
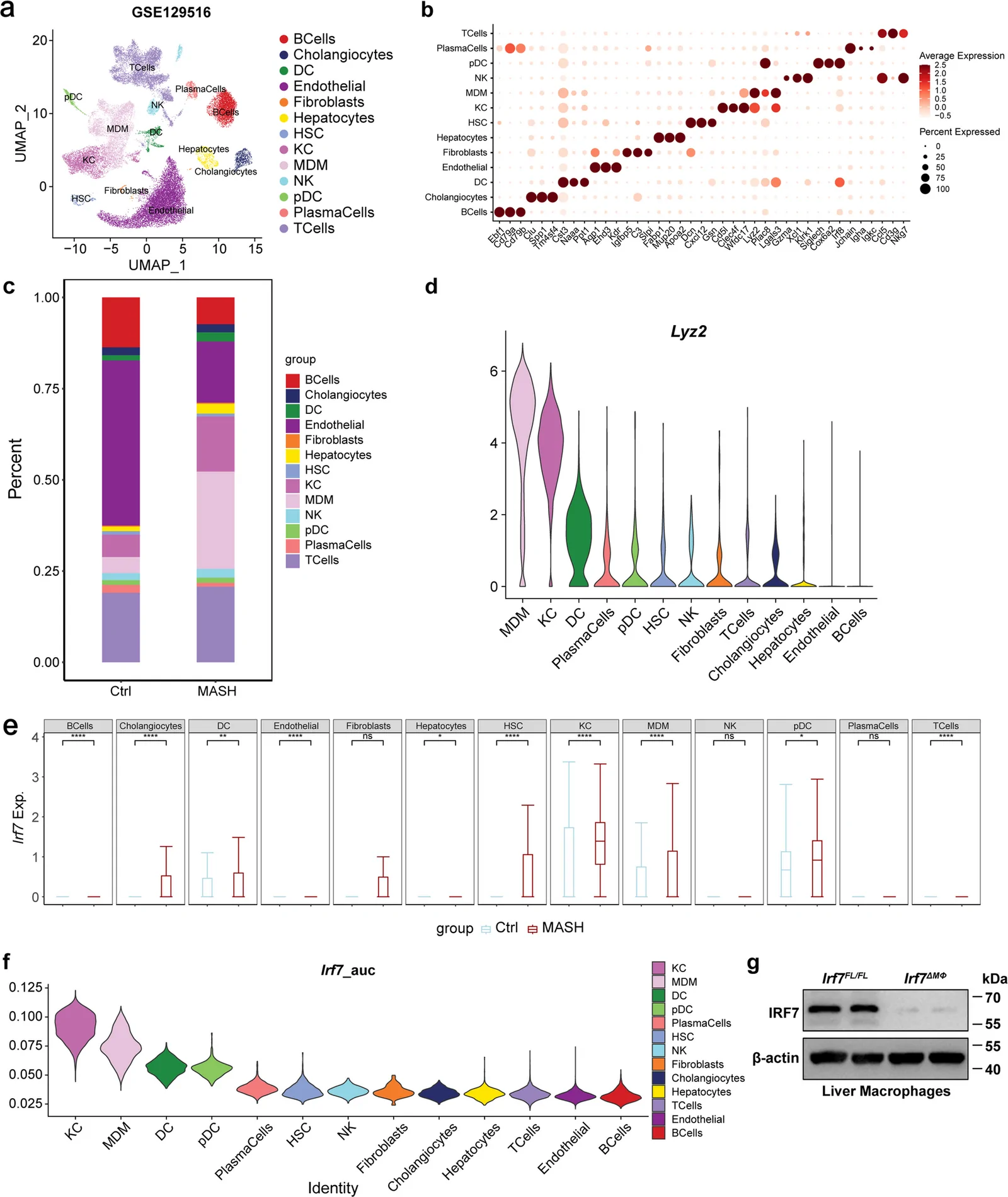
IRF7 levels are up-regulated in the macrophages. **a** A Uniform Manifold Approximation and Projection (UMAP) from 3 MASH mouse samples and 3 Chow mouse samples by analyzing GSE129516 database, colored by major cell types. **b** Heatmap showing the mean expression of characteristical 3 markers of each cell type. **c** The percentage of major cell types between the Chow and MASH tissues. **d** The RNA expression level of *Lyz2* in different cell types. **e** The RNA expression level of *Irf7* in the major cell types between the Chow and MASH tissues. Colors represent the sample types. **f** Box plots showing the predicted transcriptional level of *Irf7* in different cell types, colored by different cell types. **g** The protein expression levels of IRF7 in macrophages from *Irf7*^*fl/fl*^ and *Irf7*.^*ΔMf*^ mice. Data are presented as means ± SDs, and statistical significance was determined using Student’s t-test or ANOVA; *P < 0.05; **P < 0.01; ****P < 0.0001

**Fig. 3:**
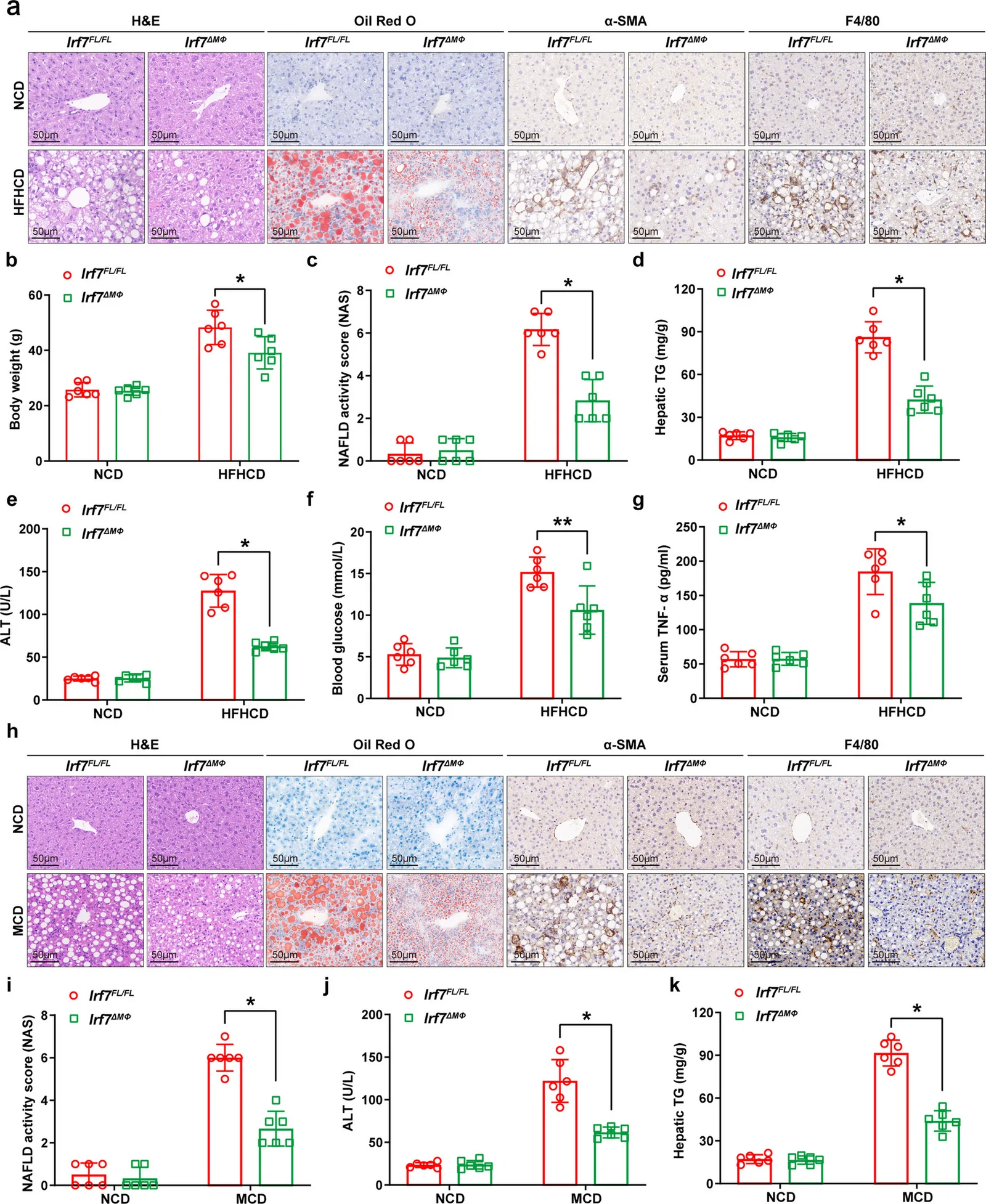
Macrophage-specific *Irf7* deficiency attenuates MASH in mice. **a-c** Male *Irf7*^*fl/fl*^ and *Irf7*^*ΔMϕ*^ mice were fed an HFHCD diet to induce experimental MASH. Liver tissues were analyzed using H&E, Oil Red O staining, and α-SMA immunohistochemistry, scale bar = 50 µm. Data represent 6 mice/group. **d** NAS, **e** serum ALT, and **f** hepatic TG were measured in *Irf7*^*fl/fl*^ and *Irf7*^*ΔMϕ*^ mice on an HFHCD diet; *n* = 6/group. **g-i** Similarly, male *Irf7*^*fl/fl*^ and *Irf7*^*ΔMϕ*^ mice were fed an MCD diet to induce experimental MASH. Liver samples underwent Oil Red O staining and α-SMA immunohistochemical analysis, scale bar = 50 µm. Data are representative of 6 mice/group. **j** NAS, **k** serum ALT, and **l** hepatic TG were assessed in *Irf7*^*fl/fl*^ and *Irf7*^*ΔM*^. ^*ϕ*^ mice on an MCD diet; *n* = 6/group. Data are presented as means ± SDs, and statistical significance was determined using Student’s t-test or ANOVA; *P < 0.05

To investigate the involvement of macrophage *Irf7* in MASH, we analyzed the scRNA-seq data of the livers from MASH mice (GSE129516). Thirteen main cell types expressing known marker genes were identified including B cells (early B cell factor 1, CD79A antigen, CD79B antigen) (*Ebf1*, *Cd79a*, *Cd79b*), cholangiocytes (Clusterin, secreted phosphoprotein 1, transmembrane 4 superfamily member 4) (*Clu*, *Spp1*, *Tm4sf4*), dendritic cells (DC) (cystatin C, N-acylethanolamine acid amidase, palmitoyl-protein thioesterase 1) (*Cst3*, *Naaa*, *Ppt1*), endothelial cells (aquaporin 1, EH-domain containing 3, kinase insert domain protein receptor) (*Aqp1*, *Ehd3*, *Kdr*), fibroblasts (insulin-like growth factor binding protein 5, complement component 3, secretory leukocyte peptidase inhibitor) (*Igfbp5*, *C3*, *Slpi*), hepatocytes (fatty acid binding protein 1, major urinary protein 20, apolipoprotein A-II) (*Fabp1*, *Mup20*, *Apoa2*), hepatic stellate cells (HSCs) (decorin, C-X-C motif chemokine ligand 12, gelsolin) (*Dcn*, *Cxcl12*, *Gsn*), KC (CD5 antigen-like, C-type lectin domain family 4, member f, WAP four-disulfide core domain 17) (*Cd5l*, *Clec4f*, *Wfdc17*), MDM (lysozyme 2, placenta-specific 8, lectin, galactose binding, soluble 3) (*Lyz2*, *Plac8*, *Lgals3*), natural killer cells (NK) (granzyme A, chemokine (C motif) ligand 1, killer cell lectin-like receptor subfamily K, member 1) (*Gzma*, *Xcl1*, *Klrk1*), plasmacytoid dendritic cells (pDC) (sialic acid binding Ig-like lectin H, cytochrome c oxidase subunit 6A2, interferon regulatory factor 8) (*Siglech*, *Cox6a2*, *Irf8*), plasma cells (immunoglobulin joining chain, immunoglobulin heavy constant alpha, immunoglobulin kappa constant) (*Jchain*, *Igha*, *Igkc*), and T cells (C–C motif chemokine ligand 5, CD3 antigen, gamma polypeptide, natural killer cell group 7 sequence) (*Ccl5*, *Cd3g*, *Nkg7*) (Fig. 2a, b). Further analysis demonstrated that the percentage of infiltrating macrophages significantly increased in MASH tissues compared with the Chow (Fig. 2c). Moreover, *Lyz2* mainly expressed in infiltrating macrophages and Kupffer cells (Fig. 2d). The RNA expression level of *Irf7* in infiltrating macrophages and Kupffer cells significantly increased in MASH tissues compared with the Chow (Fig. 2e) and transcriptional level of *Irf7* were higher in infiltrating macrophages and Kupffer cells than other cell types (Fig. 2f). Therefore, our study developed macrophage-specific *Irf7* knockout mice with Lyz2-Cre system (Fig. 2g). We developed macrophage-specific *Irf7* knockout mice by crossbreeding *Irf7*^*fl/fl*^ mice with *Lyz2-Cre* mice to examine whether the conditional deletion of *Irf7* in macrophages alleviates MASH in these mice. The results demonstrated that *Irf7*^*ΔMϕ*^ mice were significantly protected against MASH, as evidenced by mitigated liver injury with improved liver architecture and reduced Oil red O, α-SMA staining and F4/80 inflammatory cell infiltration in liver samples from HFHCD-fed *Irf7*^*ΔMϕ*^ mice compared to *Irf7*^*fl/fl*^ mice fed the HFHCD diet (Fig. 3a). Moreover, *Irf7*^*ΔMϕ*^ mice fed with HFHCD displayed significantly lower body weight, less NAFLD activity scores (NAS), hepatic triglycerides (TG), lower serum ALT, blood glucose, TNF-α, serum GTT and ITT levels compared to *Irf7*^*fl/fl*^ mice fed with HFHCD (Fig. 3b-g and S2). Mitigated liver injury and reduced Oil red O, α-SMA staining and F4/80 inflammatory cell infiltration were observed in MCD-fed *Irf7*^*ΔMϕ*^ mice relative to MCD-fed *Irf7*^*fl/fl*^ mice (Fig. 3h). Furthermore, significant reductions in NAS scores, serum ALT and hepatic TG levels were observed in *Irf7*^*ΔMϕ*^ mice compared to *Irf7*^*fl/fl*^ mice under MCD diet conditions (Fig. 3i-k). These findings demonstrate that macrophage-specific *Irf7* deficiency attenuates MASH progression.

### IRF7 regulates the macrophage type I interferon signaling loop

**Fig. 4:**
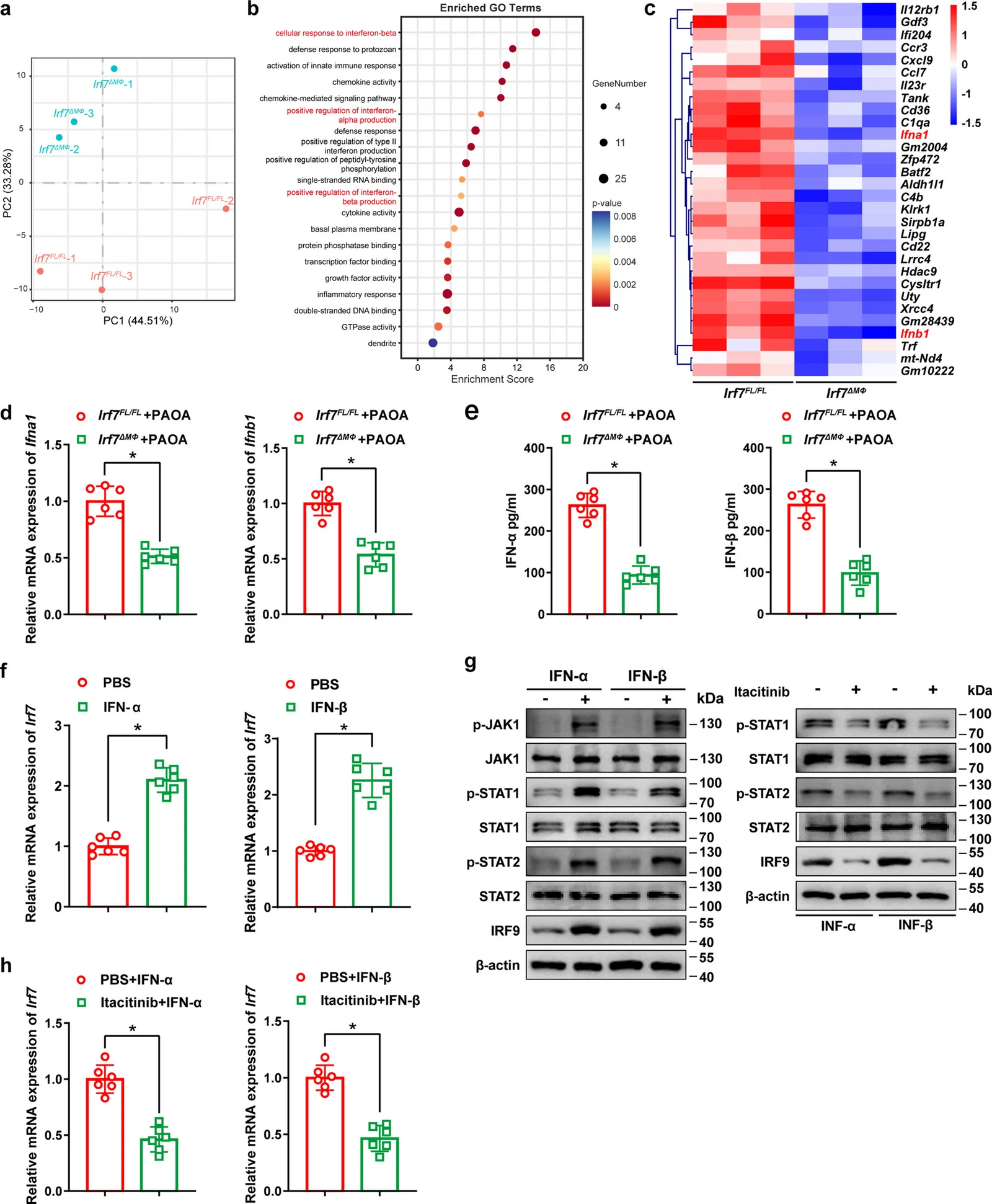
IRF7 regulates the macrophage type I interferon signaling loop. **a** Principal component analysis of transcriptome RNA-seq data on the *Irf7*^*ΔMϕ*^ and *Irf7*^*fl/fl*^ BMDMs. **b** Enriched GO terms in stimulated *Irf7*^*ΔMϕ*^ BMDMs compared to *Irf7*^*fl/fl*^ BMDMs. **c** Heatmap highlighting the downregulation of *Ifna1* and *Ifnb1* genes associated with the IFN-I response. **d** Expression levels of *Ifna1* and *Ifnb1* in BMDMs from *Irf7*^*ΔMϕ*^ and *Irf7*^*fl/fl*^ mice under PAOA-treated and untreated conditions. **e** Cytokine concentrations of IFN-α and IFN-β in the supernatant of LPS-primed BMDMs from *Irf7*^*ΔMϕ*^ and *Irf7*^*fl/fl*^ mice, with or without PAOA treatment. **f** Expression levels of *Irf7* in BMDMs derived from *Irf7*^*fl/fl*^ mice treated with IFN-α or IFN-β. **g** Protein levels of p-JAK1, JAK1, p-STAT1, STAT1, p-STAT2, STAT2, and IRF9 in BMDMs from *Irf7*^*fl/fl*^ mice treated with IFN-α or IFN-β, with or without itacitinib. **h** *Irf7* expression in LPS-primed, IFN-α- or IFN-β-treated BMDMs from *Irf7*.^*fl/fl*^ mice, with or without itacitinib treatment. Data are presented as means ± SDs, and statistical significance was determined using Student’s t-test or ANOVA; *P < 0.05; **P < 0.01

To investigate the role of IRF7 in MASH, LPS-primed bone marrow-derived macrophages (BMDMs) from either *Irf7*^*fl/fl*^ or *Irf7*^*ΔMϕ*^ mice were treated with palmitic acid/oleic acid (PAOA) for 48 h. Following treatment, Principal component analysis (PCA) was performed with transcriptome RNA-seq data on the *Irf7*^*fl/fl*^ and *Irf7*^*ΔMϕ*^ BMDMs (Fig. 4a). GO analysis revealed that these genes were primarily enriched in interferon-mediated immune responses (Fig. 4b). RNA sequencing and mRNA expression data revealed significantly lower IFN-α (*Ifna1*) and IFN-β (*Ifnb1*) expression in activated *Irf7*^*ΔMϕ*^ BMDMs compared to *Irf7*^*fl/fl*^ BMDMs (Fig. 4c, d). ELISA analysis of the secreted protein levels of IFN-α and IFN-β confirmed these findings (Fig. 4e). Previous studies have demonstrated that IFN-α and IFN-β might bind to the IFN-I receptor (IFNAR) to activate JAK/STAT signaling and promote the binding of STAT1/STAT2 with IRF9, eventually inducing IRF7 expression [12, 24]. Our results demonstrated that stimulating LPS-primed and PAOA-treated *Irf7*^*fl/fl*^ or *Irf7*^*ΔMϕ*^ BMDMs with IFN-α or IFN-β further promoted macrophage *Irf7* expression (Fig. 4f) by activating JAK/STAT signaling and promoting the binding of STAT1/STAT2 with IRF9, eventually inducing IRF7 expression (Fig. 4g). The use of the JAK1 inhibitor itacitinib revealed that IFNAR activation modulated STAT1/STAT2/IRF9 signaling via JAK/STAT signaling, which ultimately promoted IRF7 expression (Fig. 4g, h). These results collectively demonstrate that IRF7 regulates the macrophage type I interferon signaling loop.

### Macrophage IRF7 promotes hepatocyte lipid accumulation and pro-inflammatory cytokine expression by regulating the macrophage inflammatory response

**Fig. 5:**
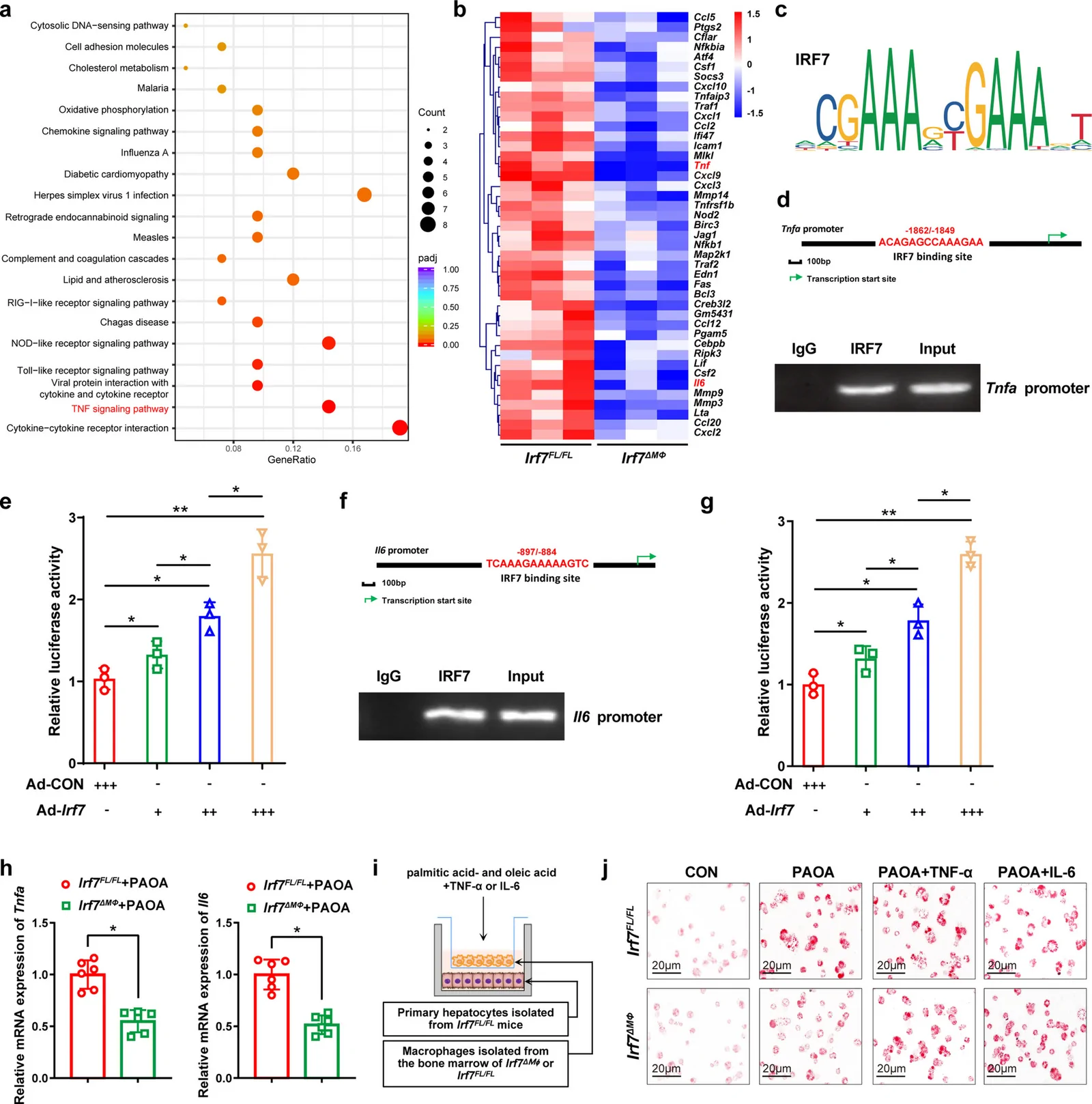
Macrophage IRF7 promotes hepatocyte lipid accumulation by regulating the macrophage inflammatory response. **a** KEGG pathway enrichment analysis illustrating pathways associated with genes downregulated in stimulated *Irf7*^*ΔMϕ*^ BMDMs compared to those in *Irf7*^*fl/fl*^ BMDMs. **b** Heatmap clustering 43 downregulated genes implicated in the TNF signaling pathway. **c** The motif of the IRF7 binding site was identified via a previously published ChIP-seq analysis of IRF7. **d** Diagram of the predicted IRF7 binding site in the *Tnfa* promoter region. ChIP assays using anti-IRF7 or IgG antibodies were performed on BMDMs. **e** Luciferase activity was measured in BMDMs that were co-transfected with a *Tnfa* promoter luciferase reporter plasmid and infected with escalating MOIs of Ad-*Irf7* or Ad-CON (*n* = 3/group). **f** Schematic of the predicted IRF7 binding site within the *Il6* promoter. ChIP assays using anti-IRF7 or IgG antibodies were conducted on BMDMs, with data representative of three experiments. **g** Luciferase activity was measured in BMDMs that were co-transfected with an *Il6* promoter luciferase reporter plasmid and infected with escalating MOIs of Ad-*Irf7* or Ad-CON (*n* = 3/group). **h** Expression levels of *Tnfa* and *Il6* were analyzed in BMDMs derived from *Irf7*^*ΔMϕ*^ or *Irf7*^*fl/fl*^ mice treated with or without PAOA. **i** Diagram showing coculture of hepatocytes from *Irf7*^*fl/fl*^ mice with BMDMs from *Irf7*^*ΔMϕ*^ or *Irf7*^*fl/fl*^ mice with or without of TNF-α, IL-6, and PAOA. **j** The accumulation of lipids in *Irf7*^*fl/fl*^ hepatocytes cocultured with *Irf7*^*ΔMϕ*^ or *Irf7*.^*fl/fl*^ macrophages in PAOA-containing medium, analyzed via Oil Red O staining, scale bar = 20 µm. Data are presented as means ± SDs, with statistical significance assessed using Student’s t-test or ANOVA; *P < 0.05; **P < 0.01

To investigate whether IRF7-driven macrophage inflammation influences hepatic lipid homeostasis, we first analyzed RNA sequencing data. The results showed that the TNF signaling pathway was significantly suppressed in *Irf7*^*ΔMϕ*^ BMDMs versus *Irf7*^*fl/fl*^ BMDMs primed with LPS, following 48 h of PAOA exposure (Fig. 5a). Downregulated genes analysis of TNF signaling pathway revealed that several key proinflammatory genes, including *Tnfa* and *Il6*, were significantly less expressed in the LPS-primed *Irf7*^*ΔMϕ*^ BMDMs than in *Irf7*^*fl/fl*^ BMDMs exposed to PAOA (Fig. 5b). Further bioinformatics analysis identified a putative IRF7 binding site (−1862/−1849) in the murine *Tnfa* promoter, which was validated through chromatin immunoprecipitation (ChIP) analysis (Fig. 5c, d). We also found that IRF7 activated *Tnfa* transcription in a concentration-dependent manner, as shown by the elevated *Tnfa*-luciferase reporter activity in BMDMs primed with LPS and co-transfected with pGL3-*Tnfa*-luciferase and Ad-*Irf7* (Fig. 5e). Additionally, bioinformatics analysis of the *Il6* promoter demonstrated another potential IRF7 binding site (−897/−884), which was validated by ChIP (Fig. 5f). Similarly, IRF7 stimulated *Il6* transcription in a concentration-dependent manner, as shown by the elevated *Il6*-luciferase reporter expression in BMDMs primed with LPS and co-transfected with pGL3-*Il6*-luciferase and Ad-*Irf7* (Fig. 5g). We then assessed the gene expression of *Tnfa* and *Il6* in *Irf7*^*fl/fl*^ or *Irf7*^*ΔMϕ*^ BMDMs primed with LPS and incubated with PAOA, finding that macrophage-specific *Irf7* deletion significantly suppressed *Tnfa* and *Il6* expression (Fig. 5h). To evaluate the effects of TNF-α and IL-6 derived from macrophages on steatohepatitis, we cocultured LPS-primed *Irf7*^*ΔMϕ*^ or *Irf7*^*fl/fl*^ BMDMs with primary *Irf7*^*fl/fl*^ hepatocytes (Fig. 5i). The results showed that macrophage-specific *Irf7* knockout markedly decreased hepatocyte lipid deposition and inflammatory responses (Fig. 5j and S3a). These results demonstrate that IRF7 promotes hepatocyte lipid accumulation and pro-inflammatory cytokine expression by targeting macrophage inflammatory genes and regulating the macrophage inflammatory response.

### Macrophage IRF7 promotes HSC activation by interacting with SMAD3 to regulate Tgfb1 transcription

**Fig. 6:**
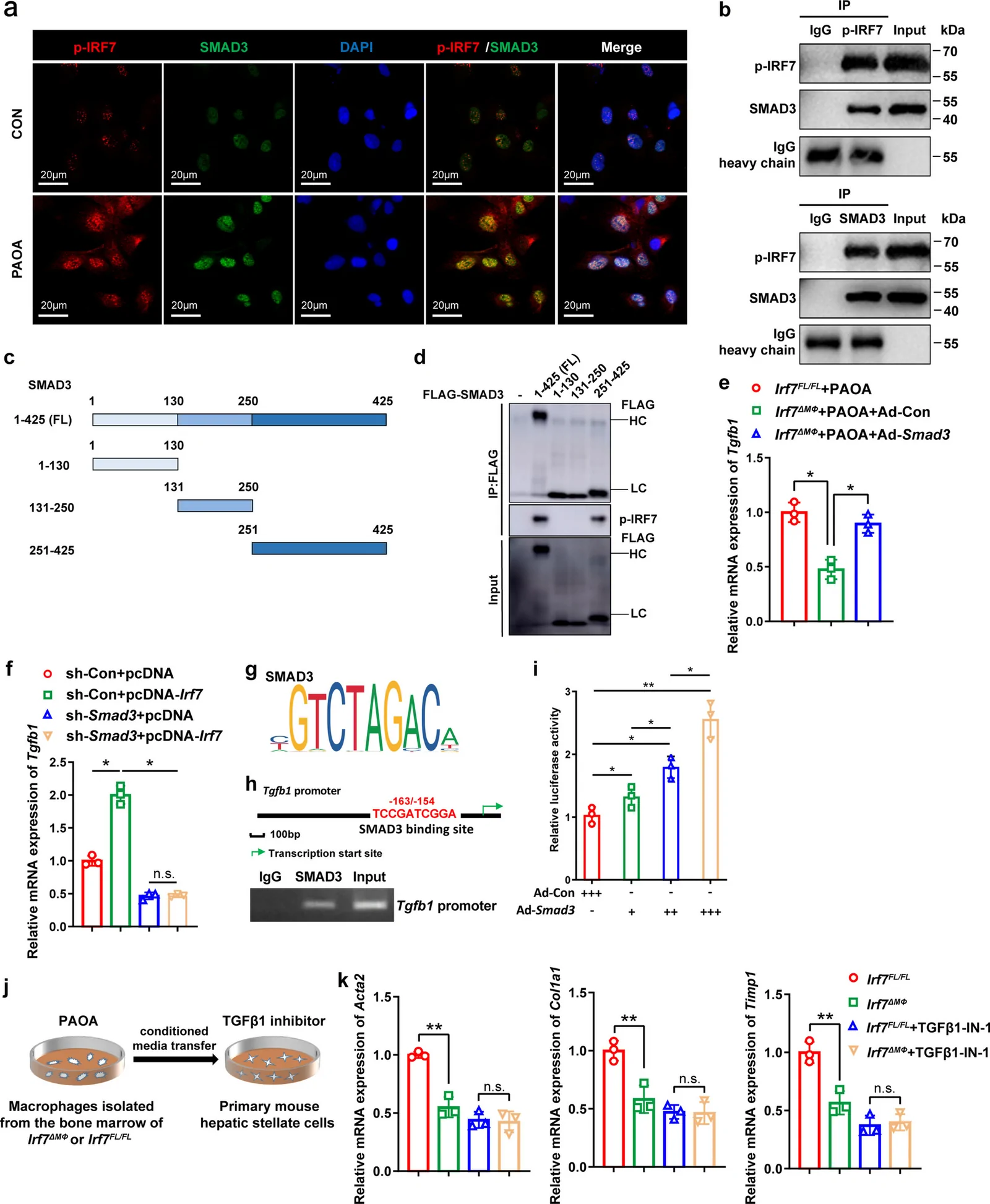
Macrophage IRF7 promotes HSC activation by interacting with SMAD3 to regulate *Tgfb1* transcription. **a** Dual immunofluorescence staining of WT BMDMs primed with LPS, treated with or without PAOA, using anti-p-IRF7 and anti-SMAD3 antibodies. Data represent three independent experiments, scale bar = 20 µm. **b** Immunoprecipitation analysis of p-IRF7 and SMAD3 in LPS-primed WT BMDMs stimulated with PAOA. **c** Schema for generating deletion mutant constructs encoding Flag-labeled SMAD3 truncates lacking specific regions. **d** Deletion mutants and a full-length SMAD3 encoding construct as well as an empty vector control was co-expressed in BMDMs. Pull-down with anti-Flag gels and subsequent immunoblotting revealed which SMAD3 variants could interact with p-IRF7. HC, high chains of antibodies used for IP. LC, low chains of antibodies used for IP. **e** Expression levels of *Tgfb1* were measured in LPS-primed BMDMs from WT mice, treated with PAOA, and infected with Ad-CON or Ad-*Smad3*. **f** Expression levels of *Tgfb1* were measured in BMDMs from *Irf7*^*ΔMϕ*^ or *Irf7*.^*fl/fl*^ mice that were primed with LPS, treated with PAOA, and transfected with sh-Ctrl or sh-*Smad3* and pcDNA or pcDNA-*Irf7*. **g** SMAD3 binding motif identified from a ChIP-seq analysis of SMAD3 that was previously published. **h** Diagram illustrating the predicted SMAD3 binding site within the *Tgfb1* promoter. ChIP assays were performed on BMDMs using anti-SMAD3 antibody or IgG, with data representative of three experiments. **i** The luciferase activity was measured in BMDMs that were co-transfected with a *Tgfb1* promoter luciferase reporter plasmid and infected with escalating MOIs of Ad-*Smad3* or Ad-CON (*n* = 3/group). **j** Conditioned medium from BMDMs that were stimulated with PAOA, primed with LPS were used to treat primary mouse hepatic stellate cells, with or without TGF-β1 (8 ng/ml for 24 h). **k** qRT-PCR were employed to assess the expression levels of *Acta2*, *Col1a1*, and *Timp1* in treated HSCs (*n* = 3/group). Data are presented as means ± SDs, statistical significance was assessed using Student’s t-test or ANOVA; *P < 0.05; **P < 0.01; ***P < 0.001

A previous study identified IRF7 as a protein that interacts with SMAD3, a critical mediator TGF-β1 signaling involved in collagen synthesis in systemic sclerosis [20]. Our previous study demonstrated the important regulatory effect of macrophages on HSC activation [9, 10, 25]. To examine the specific role of macrophage IRF7 in regulating HSC activation during MASH, we performed several experiments. Dual immunofluorescence staining revealed that IRF7 activation increased and that activated IRF7 strongly colocalized with SMAD3 in stimulated macrophages, especially in the nucleus (Fig. 6a). Coimmunoprecipitation assays revealed that activated IRF7 bound with SMAD3 in stimulated macrophages (Fig. 6b). To map the domains mediating the interaction between SMAD3 and p-IRF7, a series of deleting mutants were expressed in BMDMs (Fig. 6c, d). Co-IP assays (Fig. 6d) showed that the region (amino acids 251–425) of SMAD3 bound to p-IRF7. To explore whether IRF7 regulates *Tgfb1* transcription via SMAD3 signaling, we overexpressed SMAD3 in stimulated macrophages and found that SMAD3 overexpression in *Irf7*^*ΔMϕ*^ macrophages promoted *Tgfb1* expression (Fig. 6e). Subsequent experiments demonstrated that *Smad3* knockdown in *Irf7*^*fl/fl*^ macrophages significantly inhibited *Tgfb1* expression, whereas overexpressing *Irf7* did not increase *Tgfb1* transcription (Fig. 6f). The findings suggest that IRF7 modulates *Tgfb1* transcription through interaction with SMAD3 signaling. To further elucidate the mechanism by which SMAD3 influences *Tgfb1* transcription, bioinformatics analysis of the murine *Tgfb1* promoter revealed a potential SMAD3 binding site (−163/−154). This prediction was validated through ChIP assay results (Fig. 6g, h). SMAD3 stimulated *Tgfb1* transcriptional activity in a concentration-dependent manner, as shown by the elevated in *Tgfb1*-luciferase reporter gene expression in BMDMs stimulated with LPS and co-transfected with the pGL3-*Tgfb1*-luciferase vector and Ad-*Smad3* (Fig. 6i). To confirm the impact of macrophage IRF7 on HSC activation, primary mouse HSCs were cultured with or without TGF-β1 (8 ng/ml) in the presence of CM from LPS-primed *Irf7*^*fl/fl*^ or *Irf7*^*ΔMϕ*^ BMDMs treated with PAOA for 48 h (Fig. 6j). Gene expression analysis of fibrotic markers in cocultured HSCs revealed that macrophage *Irf7* knockout significantly inhibited HSCs activation (Fig. 6k). In summary, these results indicate that macrophage IRF7 promotes HSC activation via interaction with SMAD3 to regulate *Tgfb1* transcription.

### IRF7 is regulated by HUWE1 to modulate macrophage inflammatory responses

**Fig. 7:**
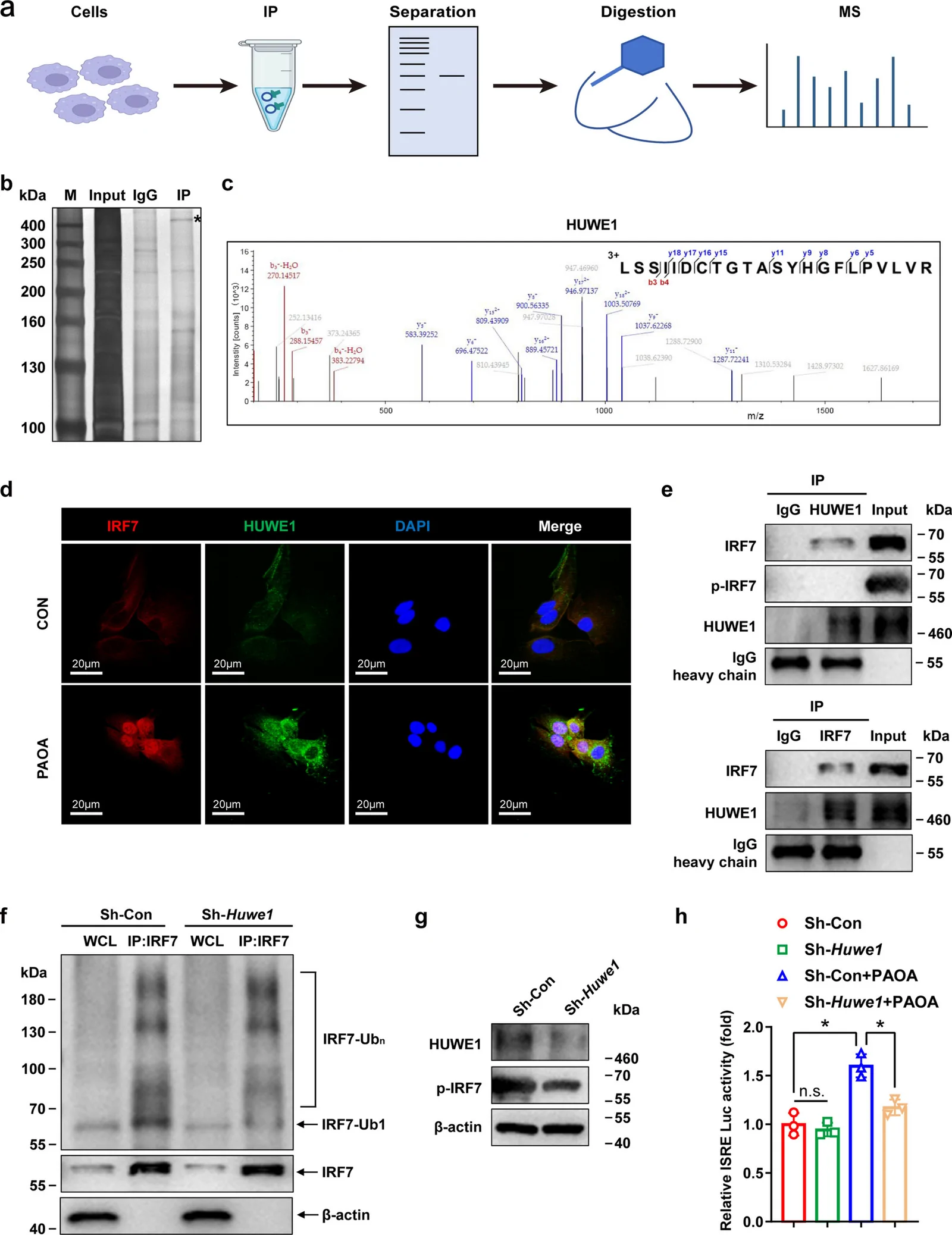
IRF7 is regulated by HUWE1 to modulate macrophage inflammatory responses. **a** Schematic diagram of the scheme for identifying proteins interacting with IRF7 in macrophages based on mass spectrometry. **b** Coimmunoprecipitation (Co-IP) complexes were visualized by Coomassie blue staining, with lane 4 showing the eluted protein. **c** Mass spectrometry was utilized to identify the peptide sequences of the HUWE1 protein within Co-IP complexes. **d** Dual immunofluorescence staining of LPS-primed BMDMs treated with PAOA, using anti-IRF7 and anti-HUWE1 antibodies, scale bar = 20 µm. **e** Co-IP assays revealed that HUWE1 bound to IRF7 in treated BMDMs. **f** Analysis of IRF7 ubiquitylation in activated BMDMs treated with sh-Con or sh-*Huwe1*. Prior to stimulation with PAOA, cells were pretreated with MG132 at a concentration of 10 µM for 3 h. **g** Protein levels of HUWE1 and p-IRF7 in activated BMDMs subjected to sh-Con or sh-*Huwe1* treatment. **h** The luciferase activity driven by interferon-stimulated response elements (ISREs) was measured in WT BMDMs with or without PAOA and sh-*Huwe1* treatment. Data are shown as means ± SDs, with statistical significance determined using Student’s t-test or ANOVA; *P < 0.05

To investigate the mechanism by which IRF7 regulates macrophage activation during MASH, immunoprecipitation (IP) assays coupled with mass spectrometry screening were conducted to identify proteins interacting with IRF7 in macrophages (Fig. 7a). Particular attention was given to evaluating the potential direct interaction between HUWE1 and IRF7 (Fig. 7b, c). Confocal immunofluorescence analysis demonstrated that IRF7 colocalized with HUWE1 mainly in the cytoplasm (Fig. 7d). Furthermore, cell lysates underwent immunoprecipitation using either anti-IRF7 or anti-HUWE1 antibodies, followed by immunoblotting analysis of the Co-IP complexes. The findings indicated that IRF7, but not p-IRF7, interacted with HUWE1 in macrophages (Fig. 7e). Beyond their physical interaction, HUWE1 facilitated the ubiquitination of IRF7. Ubiquitination analysis, followed by IRF7 immunoprecipitation, revealed the presence of several slower-migrating bands in stimulated macrophages, suggesting the formation of monoubiquitin and polyubiquitin chains (Fig. 7f). The level of monoubiquitin, especially that of IRF7-Ub1, was drastically lower in *Huwe1*-knockdown macrophages than in control macrophages (Fig. 7f). In addition to exhibiting relatively greater monoubiquitination, stimulated *Huwe1*-knockdown macrophages presented lower levels of p-IRF7 (Fig. 7g) and weaker transcriptional activity of the ISRE, which indicated lower type I interferon expression (Fig. 7h). These results indicate that IRF7 is regulated by HUWE1 to modulate macrophage inflammatory responses.

### Macrophage-specific Irf7 knockdown and pharmacological intervention attenuates MASH progression

**Fig. 8:**
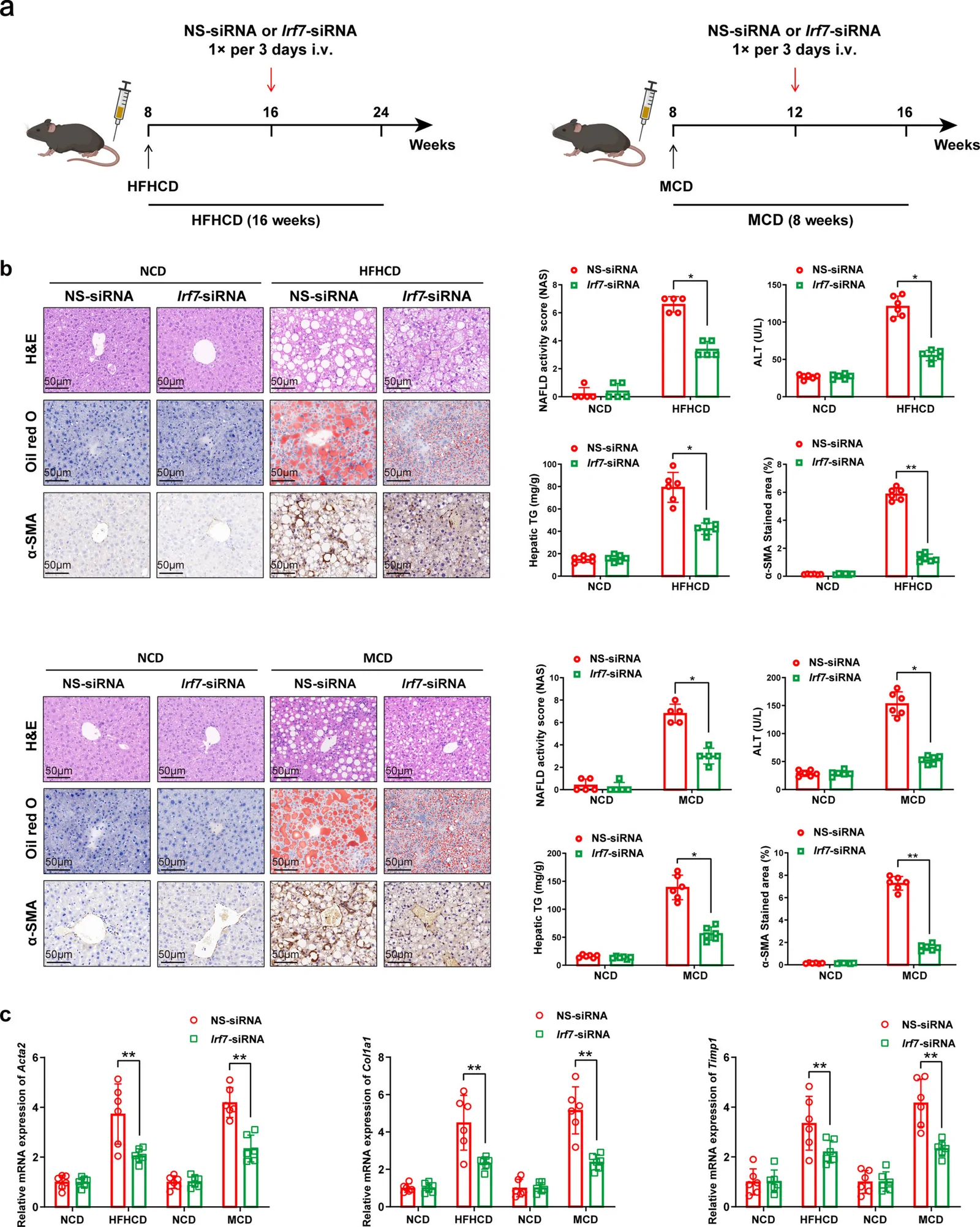
Macrophage-specific *Irf7* knockdown attenuates MASH progression. **a** Diagram illustrating the protocol for administering *Irf7*-siRNA to mice with HFHCD- or MCD-induced MASH in the experiments described in **b**. PBS served as the control. **b** NS-siRNA or *Irf7*-siRNA was combined with mannose-conjugated polymers 4 h before tail vein injection. WT male mice fed either an HFHCD or MCD diet to induce experimental MASH were injected with *Irf7*-siRNA, and their livers were subsequently collected for H&E staining, Oil Red O staining, and α-SMA immunohistochemical analysis (scale bar = 50 µm). NAS, serum ALT, and hepatic TG were measured in HFHCD-fed and MCD diet-fed mice treated with NS-siRNA or *Irf7*-siRNA, and the α-SMA-positive areas were quantified (*n* = 6 mice/group). **c** qRT-PCR was performed to assess the expression levels of *Acta2*, *Col1a1*, and *Timp1* in liver tissues from WT mice with HFHCD- or MCD-induced MASH injected with NS-siRNA or *Irf7*-siRNA (*n* = 6 mice/group). Data are presented as means ± SDs, and statistical analysis was performed using ANOVA; *P < 0.05; **P < 0.01

To test the in vivo effect of macrophage *Irf7* knockdown for future clinical application, we employed a mannose-based in vivo delivery system for *Irf7* siRNA that selectively targets macrophages through the expression of a mannose-specific membrane receptor [26]. This system was used to disrupt IRF7 in the liver of WT mice fed either an HFHCD diet or an MCD diet (Fig. 8a). The knockdown efficiency in different liver cell types after *Irf7* siRNA administration, which demonstrated that macrophage *Irf7* expression was downregulated significantly and specifically (Fig. S4a, b). *Irf7* siRNA alleviated MASH progression, as evidenced by decreased Oil red O and α-SMA staining in liver tissue (Fig. 8b), reduced serum GTT and ITT levels (Fig. S5). Furthermore, the expression of profibrogenic markers such as *Acta2*, *Col1a1*, and *Timp1* was downregulated following the administration of *Irf7* siRNA. (Fig. 8c). Furthermore, to explore the translational significance of above findings, clinical management strategy with GLP-1 receptor agonist (liraglutide) was used to demonstrated the effect of pharmacological intervention. The results indicated that injected with liraglutide alleviated MASH progression, as evidenced by decreased Oil red O and α-SMA staining in liver tissue (Fig. S6a), as well as less NAFLD activity scores, TG, lower serum ALT (Fig. S6b). Moreover, the expression of profibrogenic markers such as *Acta2*, *Col1a1*, and *Timp1* was downregulated following the administration of liraglutide (Fig. S6c). Most importantly, liraglutide administration remarkably suppressed *Irf7* expression. These results suggest that *Irf7* knockdown and pharmacological intervention notably alleviates MASH progression.

## Discussion

In this study, we identified the reduction of IRF7 in macrophage as an innovative therapeutic target for MASH. Experiments in vivo indicated that IRF7 significantly expressed in the liver tissues of MASH patients and that macrophage-specific *Irf7* knockout attenuated MASH in mice. Furthermore, subsequent experiments demonstrated that *Irf7* knockout suppressed macrophage type I IFN-dependent immune responses. In addition, IRF7 targeted the promoter of inflammatory genes (*Tnfa* and *Il1b*) and promoted proinflammatory gene transcription. Moreover, IRF7 bound to SMAD3 and targeted the *Tgfb1* promoter, thus promoting *Tgfb1* transcription. Ultimately, IRF7 enhanced the secretion of proinflammatory and profibrotic cytokines, leading to hepatic steatosis and activation of HSC. Additionally, the ubiquitin ligase E3 HUWE1 bound to IRF7, increasing IRF7 monoubiquitination and entry into the nucleus to promote downstream transcription. IRF7 disruption blocked MASH progression. These results highlight the potential of targeting IRF7 or its regulatory pathways to develop effective therapies.

Macrophage heterogeneity plays a crucial role in hepatic diseases, including MASH [27]. Liver macrophages are mainly classified into two major categories: resident kupffer cells (KCs) and infiltrating myeloid-derived macrophages (BMDMs), with distinct differences in origin, localization, phenotypic characteristics and functions. KCs are the major resident macrophage population in the steady-state liver, maintaining their numbers through self-renewal. The elevated *Irf7* expression in Kupffer cells may promote the activation of pro-inflammatory signaling pathways. However, during MASH pathogenesis, KCs undergo massive apoptosis and depletion due to lipotoxicity, oxidative stress, and ferroptosis, resulting in severe functional exhaustion. In contrast, circulating bone marrow-derived monocytes are extensively recruited into the liver via the CCL2/CCR2 chemokine axis, differentiate into BMDMs, and gradually replace KCs as the predominant hepatic macrophage population in progressive MASH, which lays the foundation for their dominant role in disease progression [27]. Therefore, our study mainly focus on macrophages in general. With the transition of MASH from the early stage to the middle stage, infiltrating macrophages are predominantly polarized into the pro-inflammatory macrophage subtype, which secretes cytokines such as TNF-α and IL-6, exacerbating steatosis and inflammation [28]. Our previous study demonstrated that the intimate crosstalk between macrophages and hepatocytes plays a key role in steatohepatitis, with proinflammatory macrophages potentially promoting hepatocyte adipogenesis [25]. Advanced liver fibrosis is a critical prognostic indicator for liver-related outcomes and overall mortality in MASH patients [29]. Increased expression of TGF-β1 and HSC activation are key drivers of fibrogenesis during MASH progression [30]. Our previous study also demonstrated that macrophages might secrete profibrotic cytokines, such as TGF-β1, which promote HSC activation during MASH [25]. The results indicated that macrophage-specific *Irf7* knockout attenuated MASH by targeting inflammatory gene (*Tnfa*, *Il1b*) and profibrotic gene (*Tgfb1*) promoters and inhibiting proinflammatory gene transcription, which eventually decreased proinflammatory and profibrotic cytokine secretion, leading to less hepatic steatosis and HSC activation.

Interferons (IFNs) have been thoroughly investigated, primarily in the immune response to viral infections. IFNs include 2 categories: type I IFNs (IFN-α and IFN-β) and type II IFNs (IFN-γ). Type I is synthesized by many kinds of cells defensing virus, whereas type II is secreted mostly by activated T cells and NK cells [31, 32]. Type I IFNs can be triggered in differentiated plasmacytoid dendritic cells through activation of the Toll-like receptor 9 (TLR9) subfamily [33, 34]. Our recent studies suggest that the type I IFN response is crucial for the progression of MASH [9, 10]. IRF7, a novel member of the IRF family, is ubiquitously expressed in the type I IFN response, which is fully regulated by IFN-α/β signaling. A previous study demonstrated that IRF7 was implicated in the development of metabolic abnormalities and that IRF7 deficiency prevented diet-induced obesity and insulin resistance, suggesting that IRF7 might be a new treatment target for obesity and type 2 diabetes [23]. However, the role and specific mechanisms by which macrophage IRF7 regulates MASH progression remain unknown. Our study revealed that macrophage-specific IRF7 deficiency suppressed macrophage inflammation by suppressing the type I IFN response, which ultimately suppressed the secretion of IFN-α and IFN-β by macrophages.

Posttranslational modifications of proteins regulate many biological processes, and the disruption of posttranslational modifications is related to disease. Ubiquitination is a posttranslational modification that regulates numerous biological pathways. The process requires three enzymes: enzyme E1 for ubiquitin-activating, enzyme E2 for ubiquitin-conjugating and enzyme E3, ubiquitin-ligase. E3s are crucial for selecting substrates. Evidence suggests that ubiquitin E3 ligases perform critical regulatory roles in MASH progression [35, 36]. To explore whether E3s affect IRF7 function, we employed mass spectrometry and revealed that the ubiquitin ligase E3 HUWE1 bound to IRF7. Earlier research showed that HUWE1 deficiency in hepatocytes protects against MASH by altering lipid homeostasis [37]. Another study demonstrated that HUWE1 promotes the activation of inflammasome and facilitate the host immunity against bacterial infection [38]. Furthermore, HUWE1 was shown to monoubiquitinate H2AX to promote signaling at stalled forks [39]. To clarify how HUWE1 regulates IRF7 function, we performed ubiquitination analyses and found that HUWE1 mediated the nondegradative monoubiquitination of IRF7, promoted its nuclear translocation and transcriptional activity, and triggered the upregulated expression of downstream inflammatory and profibrotic genes at the transcription level.

Despite the important role of IRF7 in MASH progression, limitations of this study should be acknowledged. First, our functional evidence is largely derived from mouse genetic models, and the causal role of macrophage IRF7 in human MASH remains to be formally validated. Although we observed correlations between hepatic IRF7 expression and human NAFLD-activity and fibrosis scores, human liver tissue analyses were correlative and cannot recapitulate the cell-specific causal effects observed in myeloid-specific *Irf7*-deficient mice. Second, we focused on macrophage-intrinsic IRF7 function and we cannot fully exclude potential contributions of IRF7 in other hepatic cell types (e.g. hepatocytes, hepatic stellate cells) in MASH. Third, while we identified HUWE1-dependent monoubiquitination as a critical post-translational modification controlling IRF7 nuclear translocation, we have not fully dissected whether other E3 ligases or deubiquitinating enzymes also modulate IRF7 activity in MASH. Finally, our therapeutic inference that IRF7 inhibition improve MASH is based on genetic knockout evidence, pharmacological targeting of IRF7 still requires further pre-clinical testing to evaluate drug delivery, efficacy and safety before clinical translation.

Overall, our study elucidates the role of IRF7 in the progression of MASH. *Irf7* deficiency in macrophages attenuated MASH by inhibiting macrophage type I IFN-dependent immune responses. Moreover, IRF7 targeted the promoter of inflammatory genes (*Tnfa* and *Il1b*) and promoted proinflammatory gene transcription. Moreover, IRF7 bound to SMAD3 and targeted the *Tgfb1* promoter, thus promoting *Tgfb1* transcription. Finally, IRF7 facilitated the production of inflammatory and fibrotic cytokines, leading to hepatic steatosis and activation of HSC. In addition, the ubiquitin ligase E3 HUWE1 bound to IRF7, increasing IRF7 monoubiquitination and entry into the nucleus to promote downstream transcription. IRF7 inhibition blocked MASH progression. These findings suggest that the suppression of IRF7 expression might be an effective therapeutic strategy for MASH management.

## Materials and methods

### Human liver samples

Liver samples were obtained from 40 MASH patients for gene expression analysis and pathological section examination. As controls, 40 samples were obtained from individuals without hepatic steatosis, who were treated for hepatic hemangioma at the Jiangsu Province Hospital (Table S1). The control group included individuals without diabetes, alcohol consumption, viral hepatitis, or other liver conditions. Two pathologists with extensive experience, unaware of the clinical NAFLD activity score (NAS), independently evaluated all liver specimens. NAS score ≥ 5 was used to classify patients as likely having MASH. Table S1 presents the clinical features of MASH patients. Ethical approval was obtained from the Institutional Ethics Committee of the First Affiliated Hospital of Jiangsu Province Hospital (2020-SRFA-288). Before liver biopsy or surgery, informed consents for tissue analysis were obtained from all participants. All investigations were carried out following government policies and the Declaration of Helsinki and Istanbul.

### Experimental animal models

Male 8-week-old WT, FloxP-*Irf7* (*Irf7*^*fl/fl*^), and Lyz2-Cre *Irf7* knockout (*Irf7*^*fl/fl*^; *Lyz2-Cre*) mice with a C57BL/6 genetic background (purchased from GemPharmatech Co., Ltd.) were utilized for the experiments. The mice were maintained in a SPF environment and provided with sterilized food and water. Hepatic fibrosis models were developed by feeding mice an HFHCD or an MCD diet. Briefly, the mice were randomly divided into four groups: one group received 16 weeks of NCD (*n* = 6–8/group), another group was fed NCD for 8 weeks (*n* = 6–8/group), a third group was fed HFHCD (Research Diets, Inc.) for 16 weeks (*n* = 6–8/group), and the final group was fed MCD (Research Diets, Inc.) for 8 weeks (*n* = 6–8/group). Liver tissues were then collected for further analysis. Following the manufacturer’s guidelines, *Irf7* siRNA or scrambled siRNA (Invitrogen, Stockholm, Sweden) was prepared with mannose-conjugated polymers (Polyplus-transfection, France) and delivered to the mice through tail vein (2 mg/kg). The sequences of *Irf7* siRNA: sense, 5’-GGGAUCCAGUUGAUCCGCAUAAGGU-3’; antisense, 5’-ACCUUAUGCGGAUCAACUGGAUCCC-3’. Upon completion of the treatment period, liver tissues were harvested. For mice with HFHCD diet, siRNA was injected every 3 days after 8 weeks of HFHCD feeding, while after 4 weeks for the MCD group. For mice with MCD diet, either saline or liraglutide (200 µg/kg, HY-P0014, MCE, Shanghai, China) was injected daily after 4 weeks for the MCD group, until the end of the animal modeling period. After in vivo siRNA administration, single-cell suspensions are separated through liver perfusion, and various cell types are sorted by gradient centrifugation. The expression changes of target genes in different cells are quantitatively detected using qRT-PCR, in order to accurately assess their knockdown efficiency in each cell type. Body weight was longitudinally monitored throughout the experimental period. Mice were weighed at regular intervals, and serial body-weight values were recorded for each animal. Daily food intake was monitored for one week by daily weighing of cage-rack food, and values were normalized to kilocalories per day (kcal/day). All animal experiments were conducted in accordance with guidelines of the IACUC of Nanjing Medical University (IACUC-2304026).

### GTT and ITT experiments

For intraperitoneal glucose tolerance test (GTT), mice were fasted overnight (12 h) with free access to water. Baseline blood glucose was measured from tail-tip venous blood using a glucometer, followed by intraperitoneal injection of glucose (2 g/kg body weight). Blood glucose levels were sequentially determined at 0, 15, 30, 60, 90, and 120 min post-injection. For insulin tolerance test (ITT), mice were fasted for 6 h before intraperitoneal administration of insulin (0.75 units/kg body weight). Blood glucose was detected at 0, 15, 30, 60, 90, and 120 min. Serum samples were collected at designated time points for insulin quantification. GTT-AUC and ITT-AUC were calculated using the trapezoidal rule.

### Cell isolation, culture and treatment

Primary hepatocytes, liver macrophages, hepatic stellate cells, liver sinusoidal endothelial cells, and cholangiocytes were extracted using the method described earlier [25, 40]. Cells were treated with PA (500 μM; Sigma Aldrich) and OA (1000 μM; Sigman Aldrich). BSA used as a carrier control. Bone marrow cells were isolated from the femurs and tibias of male mice and flushed with DMEM. The cells were cultured at a concentration of 3 × 10^6^/mL in DMEM supplemented with 10% FBS and 20 ng/mL M-CSF (Peprotech, USA) to differentiate into BMDMs. After 7 days, all adherent cells had transitioned into fully mature macrophages.

For the coculture experiments, primary hepatocytes and BMDMs primed with LPS were plated in a coculture chamber (Corning, USA). PAOA were added to the coculture system. After 24 h of treatment with TNF-α (10 ng/mL) or IL-6 (12.5 ng/mL), the hepatocytes were analyzed for lipid accumulation and inflammatory response.

BMDMs primed with LPS were stimulated with PAOA for 48 h, followed by RNA sequencing analysis. Primary mouse HSCs were isolated following the previously described protocol [10]. In brief, following portal vein cannulation, the liver was perfused for 15 min with Ca^2+^/Mg^2+^-free HBSS at 37 °C, then with HBSS containing 0.05% collagenase and Ca^2+^/Mg^2+^ at a flow rate of 10 mL/min for another 15 min. The liver, after perfusion, was chopped and filtered through a 70 μm cell strainer, followed by centrifugation at 50 × g for 3 min. After centrifuging the supernatant at 500 × g for 10 min, the pellet was resuspended in Ficoll plus Percoll (1:10, GE Healthcare) and subjected to centrifugation at 1400 × g for 17 min. HSCs were isolated from the interface and were incubated with BMDM-CM with or without TGF-β1 (8 ng/mL) for 24 h, and the activation status of HSCs was analyzed.

### RNA sequencing and analysis

LPS-primed *Irf7*^*FL/FL*^ or *Irf7*^*ΔMϕ*^ BMDMs were stimulated with PAOA for 48 h, and then *Irf7*^*FL/FL*^ or *Irf7*^*ΔMϕ*^ BMDMs were subjected to RNA sequencing analysis (*n* = 3/group). Total RNA was extracted from cells by Trizol reagent (Invitrogen) separately. The cDNA libraries were constructed for each pooled RNA sample with the NEBNext® Ultra™ RNA Library Prep Kit for Illumina® (NEB, USA) according to the manufacturers’ instructions. Pathway analysis was used to find out the significant pathway of the differential genes according to KEGG database.

### Coimmunoprecipitation and mass spectrometry

As previously described [41], specific primary antibodies were conjugated to protein A/G beads (Selleck, CN). Then the harvested cell lysates and the beads were mixed together for incubation at 4 °C overnight. After washing, immunoprecipitated proteins were subjected to the subsequent mass spectrometry (MS) analysis, also boiled with loading buffer for western blot.

### Chromatin immunoprecipitation (ChIP) assays

A ChIP Assay Kit (Abcam, MA) were used for ChIP assays. Briefly, BMDMs were fixed with formaldehyde for 10 min to stabilize protein-chromatin interactions, and the process was stopped by adding 125 mM glycine for 5 min. Cells were then rinsed with PBS and lysed in ChIP lysis buffer for 10 min. After centrifugation, nuclear pellets were isolated, resuspended in nuclear lysis buffer, and sonicated for 15 min. Purified chromatin was electrophoresed in a 1.5% agarose gel to assess the size of DNA fragment. For immunoprecipitation, the chromatin samples were incubated overnight with antibodies against IRF7 or SMAD3 (Cell Signaling Technology, USA). Normal IgG was used as a control. The antibody/chromatin samples mixed with protein A Sepharose beads were subjected to extensive washing, and eluted. DNA was purified by cross-link reversal and analyzed via PCR using primers targeting promoter regions of interest. Primer sequences are provided for SMAD3- and IRF7-associated regions. The primers for the SMAD3-responsive region of the *Tgfb1* promoter: forward: 5’-GACTGTCGGGGGCACTTAAT-3’, reverse: 5’-AGGAGACCGACGTCTGAGAG-3’. The primers for the IRF7-responsive region of the *Tnfa* promoter: forward: 5’-CACACGAGGTCCAGCTCTTT-3’, reverse: 5’-GTAGATGGGAGATGCCGTCG-3’. The primers for the IRF7-responsive region of the *Il6* promoter: forward: 5’-TTCTTCACAAGAATCAACCGGC-3’, reverse: 5’-TCTCAGGATCACAATCAGCTTCT-3’.

### Luciferase assays

*Tgfb1* promoter-, *Tnfa* promoter-, and *Il1b* promoter-luciferase reporter plasmids (*Tgfb1*, *Tnfa*, and *Il1b* luciferase) were constructed in the pGL3 luciferase vector (Promega, WI). BMDMs were transfected with these constructs and subsequently infected with adenoviral vectors encoding *IRF7*, *SMAD3*, or a control vector [42, 43]. Cells were lysed with passive lysis buffer after 48 h. A luciferase assay system (Promega, USA) was used to measure the transcriptional activity.

### Plasmid construction and lentivirus production

Expression plasmids were constructed using the pcDNA3.1-Flag vector. Full-length SMAD3 was amplified from BMDM cDNA by PCR using gene-specific primers and verified by Sanger sequencing. Site-directed mutants were generated by overlap-extension PCR or using the Fast Mutagenesis Kit V2 (Vazyme Biotech, China) in accordance with the manufacturer's instructions. Chimeric SMAD3 constructs were generated by overlap-extension PCR. All coding sequences were cloned into the pcDNA3.1 vector with the indicated epitope tags and confirmed by sequencing. For stable overexpression, the coding sequences were subcloned into the lentiviral vector pLVX containing either a puromycin resistance cassette or GFP selection marker and verified by sequencing. Lentiviral particles were generated by co-transfecting the pLVX expression plasmids together with the packaging plasmids psPAX2 and pMD2.G into HEK293T cells using polyethyleneimine (PEI; Yeasen Biotechnology, China). Viral supernatants were collected 48 h after transfection, filtered through a 0.45-μm membrane, and used to infect target cells with polybrene (8 μg/mL; Sigma-Aldrich). Stably transduced cells were selected with puromycin.

#### Co-IP of truncation mutants

BMDMs were transiently transfected with the indicated full-length or truncation constructs. 24 h after transfection, cells were harvested and lysed in IP lysis buffer supplemented with protease and phosphatase inhibitors. Cell lysates were incubated with the indicated antibodies followed by Protein A/G agarose or magnetic beads. After extensive washing, the immunocomplexes were eluted and subjected to immunoblot analysis.

#### Histological, immunohistochemical and immunofluorescence analysis

Liver sections were prepared from liver samples after fixation in formalin and paraffin embedding. H&E staining was employed for histopathological evaluation, while Oil Red O staining quantified lipid deposition. For immunohistochemistry, sections were incubated with anti-α-SMA antibodies (Cell Signaling Technology, USA), followed by HRP-linked secondary antibodies and stained with 3,3’-diaminobenzidine tetrachloride. Then nuclear counterstaining was performed with hematoxylin. Immunofluorescence staining was to analyze the expression of p-IRF7, Albumin, Desmin, CD68, CD31, and CK19 in liver tissues, and IRF7, HUWE1, p-IRF7, and SMAD3 in cells, using: anti-rabbit p-IRF7 mAbs (Affinity, CN), anti-mouse Albumin mAbs (Proteintech, USA), anti-rabbit Desmin mAbs (Abcam, UK), anti-mouse CD68 mAbs (Abcam, UK), anti-rabbit CD31 mAbs (Abcam, UK), anti-rabbit CK19 mAbs (Abcam, UK), anti-mouse IRF7 mAbs (Santacruz, USA), anti-rabbit HUWE1 pAbs (Invitrogen, USA), anti-rabbit p-IRF7 pAbs (Affinity, China) and anti-mouse SMAD3 mAbs (Invitrogen, USA), goat anti-rabbit IgG (Texas Red-conjugated), goat anti-rabbit IgG (Texas Green-conjugated), goat anti-mouse IgG (Texas Green-conjugated) (Sigma, USA), anti-mouse IgG (H + L) (Alexa Fluor® 647 Conjugate), anti-rabbit IgG (H + L) (Alexa Fluor® 488 Conjugate), anti-rabbit IgG (H + L) (Alexa Fluor® 647 Conjugate) or anti-mouse IgG (H + L) (Alexa Fluor® 488 Conjugate) (Cell Signaling Technology, USA) secondary antibodies. Nuclei were stained with DAPI, and fluorescence was visualized via a confocal microscope (Zeiss, Germany).

#### Western blot

Proteins were resolved by SDS-PAGE, transferred onto membranes, and incubated with primary antibodies: anti-p-IRF7 rabbit pAbs (Affinity, CN), anti-IRF7 mouse mAbs (Santacruz, USA), anti-HUWE1 rabbit mAbs (Invitrogen, Carlsbad, USA), anti-SMAD3, and anti-β-actin rabbit mAbs (Abcam, UK). Secondary antibodies conjugated with HRP were used for detection.

#### Ubiquitination analysis

Cells were pretreated with MG132 (Sigma‒Aldrich) before being stimulated by mixture including: LPS (10 ng/ml) + PAOA for 4 h. Immunoprecipitates were isolated from the conjugated beads and prepared for analysis.

#### Real-time qPCR

Total RNA from liver tissues, BMDMs, or HSCs was extracted using RNA Purification Kit (SHANGHAI YISHAN Biotech Co., Ltd., CN). Complementary DNA (cDNA) synthesis was performed with a kit (Vazyme Biotech Co., Ltd., CN). Quantitative real-time PCR was conducted by a LightCycler 480 system and SYBR Green Master Mix (Vazyme Biotech Co., Ltd., CN), with β-actin serving as control. Primer sequences are listed in Table S2.

#### Statistical analysis

Data are showed as means ± SDs. Statistical comparisons between groups were performed with Student’s t-test or one-way ANOVA in SPSS 22.0 software, with *P* < 0.05 considered statistically significant.

## Supplementary Information

Supplementary Material 1.

## Data Availability

All data supporting the conclusions presented in this study can be found in the main text and supplementary materials. The scRNA-seq data from the livers of diet-induced mouse MASH models analysed during the current study are available in the Gene Expression Omnibus (GEO) database with accession code GSE129516. Bulk RNA-Seq data have been deposited into the CNCB-NGDC repository with accession code CRA043177.

## References

[1] Loomba R, Friedman SL, Shulman GI. Mechanisms and disease consequences of nonalcoholic fatty liver disease. Cell. 2021;184(10):2537–64. 10.1016/j.cell.2021.04.015. PMC12168897 33989548

[2] Machado MV, Diehl AM. Pathogenesis of Nonalcoholic Steatohepatitis. Gastroenterology. 2016;150(8):1769–77. 10.1053/j.gastro.2016.02.066. PMC4887389 26928243

[3] Rivera-Esteban J, Muñoz-Martínez S, Higuera M, Sena E, Bermúdez-Ramos M, Bañares J, et al. Phenotypes of metabolic dysfunction-associated steatotic liver disease-associated hepatocellular carcinoma. Clin Gastroenterol Hepatol. 2024. 10.1016/j.cgh.2024.03.028. 38604295

[4] Horn P, Tacke F. Metabolic reprogramming in liver fibrosis. Cell Metab. 2024;36(7):1439–55. 10.1016/j.cmet.2024.05.003. 38823393

[5] Tacke F, Puengel T, Loomba R, Friedman SL. An integrated view of anti-inflammatory and antifibrotic targets for the treatment of NASH. J Hepatol. 2023;79(2):552–66. 10.1016/j.jhep.2023.03.038. PMC12164378 37061196

[6] Lee KC, Wu PS, Lin HC. Pathogenesis and treatment of non-alcoholic steatohepatitis and its fibrosis. Clin Mol Hepatol. 2023;29(1):77–98. 10.3350/cmh.2022.0237. PMC9845678 36226471

[7] Peiseler M, Schwabe R, Hampe J, Kubes P, Heikenwälder M, Tacke F. Immune mechanisms linking metabolic injury to inflammation and fibrosis in fatty liver disease - novel insights into cellular communication circuits. J Hepatol. 2022;77(4):1136–60. 10.1016/j.jhep.2022.06.012. 35750137

[8] Krenkel O, Tacke F. Liver macrophages in tissue homeostasis and disease. Nat Rev Immunol. 2017;17(5):306–21. 10.1038/nri.2017.11. 28317925

[9] Wang Q, Bu Q, Liu M, Zhang R, Gu J, Li L, et al. XBP1-mediated activation of the STING signalling pathway in macrophages contributes to liver fibrosis progression. JHEP Rep. 2022;4(11):100555. 10.1016/j.jhepr.2022.100555. PMC9520276 36185574

[10] Wang Q, Bu Q, Xu Z, Liang Y, Zhou J, Pan Y, et al. Macrophage ATG16L1 expression suppresses metabolic dysfunction-associated steatohepatitis progression by promoting lipophagy. Clin Mol Hepatol. 2024;30(3):515–38. 10.3350/cmh.2024.0107. PMC11261221 38726504

[11] Taniguchi T, Ogasawara K, Takaoka A, Tanaka N. IRF family of transcription factors as regulators of host defense. Annu Rev Immunol. 2001;19:623–55. 10.1146/annurev.immunol.19.1.623. 11244049

[12] Darnell JE Jr., Kerr IM, Stark GR. Jak-STAT pathways and transcriptional activation in response to IFNs and other extracellular signaling proteins. Science. 1994;264(5164):1415–21. 10.1126/science.8197455. 8197455

[13] Ihle JN, Kerr IM. Jaks and stats in signaling by the cytokine receptor superfamily. Trends Genet. 1995;11(2):69–74. 10.1016/s0168-9525(00)89000-9. 7716810

[14] Bluyssen AR, Durbin JE, Levy DE. ISGF3 gamma p48, a specificity switch for interferon activated transcription factors. Cytokine Growth Factor Rev. 1996;7(1):11–7. 10.1016/1359-6101(96)00005-6. 8864350

[15] Zhang XJ, Jiang DS, Li H. The interferon regulatory factors as novel potential targets in the treatment of cardiovascular diseases. Br J Pharmacol. 2015;172(23):5457–76. 10.1111/bph.12881. PMC4667854 25131895

[16] Bovolenta C, Driggers PH, Marks MS, Medin JA, Politis AD, Vogel SN, et al. Molecular interactions between interferon consensus sequence binding protein and members of the interferon regulatory factor family. Proc Natl Acad Sci U S A. 1994;91(11):5046–50. 10.1073/pnas.91.11.5046. PMC43928 8197182

[17] Feng D, Sangster-Guity N, Stone R, Korczeniewska J, Mancl ME, Fitzgerald-Bocarsly P, et al. Differential requirement of histone acetylase and deacetylase activities for IRF5-mediated proinflammatory cytokine expression. J Immunol. 2010;185(10):6003–12. 10.4049/jimmunol.1000482. PMC3233222 20935208

[18] Guo HY, He HX, Liu BS, Zhang N, Zhu KC, Zhang DC. The regulatory mechanisms of IRF7 mediated by the type I IFN signalling pathway against *Streptococcus iniae* in yellowfin seabream, *Acanthopagrus latus* (Hottuyn, 1782). Int J Biol Macromol. 2023;247:125635. 10.1016/j.ijbiomac.2023.125635. 37399879

[19] Chen H, Chew G, Devapragash N, Loh JZ, Huang KY, Guo J, et al. The E3 ubiquitin ligase WWP2 regulates pro-fibrogenic monocyte infiltration and activity in heart fibrosis. Nat Commun. 2022;13(1):7375. 10.1038/s41467-022-34971-6. PMC9712659 36450710

[20] Wu M, Skaug B, Bi X, Mills T, Salazar G, Zhou X, et al. Interferon regulatory factor 7 (IRF7) represents a link between inflammation and fibrosis in the pathogenesis of systemic sclerosis. Ann Rheum Dis. 2019;78(11):1583–91. 10.1136/annrheumdis-2019-215208. PMC7167109 31439591

[21] Carmona FD, Gutala R, Simeón CP, Carreira P, Ortego-Centeno N, Vicente-Rabaneda E, et al. Novel identification of the IRF7 region as an anticentromere autoantibody propensity locus in systemic sclerosis. Ann Rheum Dis. 2012;71(1):114–9. 10.1136/annrheumdis-2011-200275. PMC3369428 21926187

[22] Sermasathanasawadi R, Kato N, Muroyama R, Dharel N, Shao RX, Chang JH, et al. Association of interferon regulatory factor-7 gene polymorphism with liver cirrhosis in chronic hepatitis C patients. Liver Int. 2008;28(6):798–806. 10.1111/j.1478-3231.2008.01725.x. 18397234

[23] Wang XA, Zhang R, Zhang S, Deng S, Jiang D, Zhong J, et al. Interferon regulatory factor 7 deficiency prevents diet-induced obesity and insulin resistance. Am J Physiol Endocrinol Metab. 2013;305(4):E485-495. 10.1152/ajpendo.00505.2012. 23695216

[24] Ng CT, Mendoza JL, Garcia KC, Oldstone MB. Alpha and beta type 1 interferon signaling: passage for diverse biologic outcomes. Cell. 2016;164(3):349–52. 10.1016/j.cell.2015.12.027. PMC4733246 26824652

[25] Wang Q, Zhou H, Bu Q, Wei S, Li L, Zhou J, et al. Role of XBP1 in regulating the progression of non-alcoholic steatohepatitis. J Hepatol. 2022;77(2):312–25. 10.1016/j.jhep.2022.02.031. 35292349

[26] Yu SS, Lau CM, Barham WJ, Onishko HM, Nelson CE, Li H, et al. Macrophage-specific RNA interference targeting via “click”, mannosylated polymeric micelles. Mol Pharm. 2013;10(3):975–87. 10.1021/mp300434e. PMC3595119 23331322

[27] Krenkel O, Hundertmark J, Abdallah AT, Kohlhepp M, Puengel T, Roth T, et al. Myeloid cells in liver and bone marrow acquire a functionally distinct inflammatory phenotype during obesity-related steatohepatitis. Gut. 2020;69(3):551–63. 10.1136/gutjnl-2019-318382. 31076404

[28] Luo X, Li H, Ma L, Zhou J, Guo X, Woo SL, et al. Expression of STING is increased in liver tissues from patients with NAFLD and promotes macrophage-mediated hepatic inflammation and fibrosis in mice. Gastroenterology. 2018;155(6):1971-1984.e1974. 10.1053/j.gastro.2018.09.010. PMC6279491 30213555

[29] Wang S, Friedman SL. Found in translation-fibrosis in metabolic dysfunction-associated steatohepatitis (MASH). Sci Transl Med. 2023;15(716):eadi0759. 10.1126/scitranslmed.adi0759. PMC10671253 37792957

[30] Fondevila MF, Fernandez U, Heras V, Parracho T, Gonzalez-Rellan MJ, Novoa E, et al. Inhibition of carnitine palmitoyltransferase 1A in hepatic stellate cells protects against fibrosis. J Hepatol. 2022;77(1):15–28. 10.1016/j.jhep.2022.02.003. 35167910

[31] Lengyel P. Biochemistry of interferons and their actions. Annu Rev Biochem. 1982;51:251–82. 10.1146/annurev.bi.51.070182.001343. 6180680

[32] Pestka S, Langer JA, Zoon KC, Samuel CE. Interferons and their actions. Annu Rev Biochem. 1987;56:727–77. 10.1146/annurev.bi.56.070187.003455. 2441659

[33] Honda K, Yanai H, Negishi H, Asagiri M, Sato M, Mizutani T, et al. IRF-7 is the master regulator of type-I interferon-dependent immune responses. Nature. 2005;434(7034):772–7. 10.1038/nature03464. 15800576

[34] Sin WX, Yeong JP, Lim TJF, Su IH, Connolly JE, Chin KC. IRF-7 mediates type I IFN responses in endotoxin-challenged mice. Front Immunol. 2020;11:640. 10.3389/fimmu.2020.00640. PMC7176903 32373120

[35] Nikolaou KC, Godbersen S, Manoharan M, Wieland S, Heim MH, Stoffel M. Inflammation-induced TRIM21 represses hepatic steatosis by promoting the ubiquitination of lipogenic regulators. JCI Insight. 2023;8(21). 10.1172/jci.insight.164694. PMC10721265 37937648

[36] Das A, Cheng H, Wang Y, Kinch LN, Liang G, Hong S, et al. The ubiquitin E3 ligase BFAR promotes degradation of PNPLA3. Proc Natl Acad Sci U S A. 2024;121(6):e2312291121. 10.1073/pnas.2312291121. PMC10861911 38294943

[37] Feng WW, Bang S, Takacs EM, Day C, Crawford KJ, Al-Sheyab R, et al. Hepatic Huwe1 loss protects mice from non-alcoholic fatty liver disease through lipid metabolic rewiring. iScience. 2023;26(12):108405. 10.1016/j.isci.2023.108405. PMC10692727 38047073

[38] Guo Y, Li L, Xu T, Guo X, Wang C, Li Y, et al. HUWE1 mediates inflammasome activation and promotes host defense against bacterial infection. J Clin Invest. 2020;130(12):6301–16. 10.1172/jci138234. PMC7685759 33104527

[39] Choe KN, Nicolae CM, Constantin D, Imamura Kawasawa Y, Delgado-Diaz MR, De S, et al. HUWE1 interacts with PCNA to alleviate replication stress. EMBO Rep. 2016;17(6):874–86. 10.15252/embr.201541685. PMC5278616 27146073

[40] Zhuo X, Wang K, Shi Y, Wang X, Zhan H, Yu J, et al. Isolation of primary hepatocytes and non-parenchymal cells from mouse fatty liver. MethodsX. 2026;16:103888. 10.1016/j.mex.2026.103888. PMC13090693 42007404

[41] Rao J, Wang H, Ni M, Wang Z, Wang Z, Wei S, et al. FSTL1 promotes liver fibrosis by reprogramming macrophage function through modulating the intracellular function of PKM2. Gut. 2022;71(12):2539–50. 10.1136/gutjnl-2021-325150. PMC9664121 35140065

[42] Romieu-Mourez R, Solis M, Nardin A, Goubau D, Baron-Bodo V, Lin R, et al. Distinct roles for IFN regulatory factor (IRF)-3 and IRF-7 in the activation of antitumor properties of human macrophages. Cancer Res. 2006;66(21):10576–85. 10.1158/0008-5472.Can-06-1279. 17079482

[43] Kundi R, Hollenbeck ST, Yamanouchi D, Herman BC, Edlin R, Ryer EJ, et al. Arterial gene transfer of the TGF-beta signalling protein Smad3 induces adaptive remodelling following angioplasty: a role for CTGF. Cardiovasc Res. 2009;84(2):326–35. 10.1093/cvr/cvp220. PMC2761202 19570811

