# Supplementary material for: Macrophage interferon regulatory factor 7 aggravates the progression of metabolic dysfunction-associated steatohepatitis
Source: Mol Biomed. 2026 Oct 19;7:216. doi: 10.1186/s43556-026-00601-6 (PMC13680338; doi:10.1186/s43556-026-00601-6)
Supplement: Supplementary file 1 [file 43556_2026_601_MOESM1_ESM.docx]

**Macrophage interferon regulatory factor 7 aggravates the progression of metabolic dysfunction-associated steatohepatitis**

**Xuhao Ni^1,#^, Qingfa Bu^2,#^, Yuan Liang^3,#^, Bozhou Pan^1,#^, Jian Wang^1^, Qingxiang Xu^1,*^, Lei Liu^1,*^, Qi Wang^1,*^**

^1^Department of General Surgery, First Affiliated Hospital of Anhui Medical University, Hefei, China.

^2^Hepatobiliary Center, The First Affiliated Hospital of Nanjing Medical University, Nanjing, China.

^3^School of Biological Science & Medical Engineering, Southeast University, Nanjing, China

**^#^**These authors contributed equally to this paper.

**Corresponding author:** Qi Wang, E-mail: [qwang@ahmu.edu.cn](mailto:qwang@ahmu.edu.cn); Lei Liu, E-mail: [leiliu@ahmu.edu.cn](mailto:leiliu@ahmu.edu.cn); Qingxiang Xu, E-mail: [xqx@ahmu.edu.cn](mailto:xqx@ahmu.edu.cn).

**Supplementary Tables**

**Table S1. Demographics of all patient populations**

| **Clinical manifestation** | Patients with MASH | Patients with hepatic hemangioma |
| --- | --- | --- |
| Age, years (mean±SD) | 41.05±8.02 | 40.03±7.13 |
| Gender, n (M/F) | 26/14 | 9/31 |
| BMI, kg/m^2^ | 39.61±4.68 | 21.79±2.26 |
| Hyperlipidemia (%) | 27 (68) | 0 (0) |
| **Laboratory tests** (mean±SD) |  |  |
| ALT, U/L | 109.09±46.65 | 20.25±5.28 |
| AST, U/L | 83.11±32.17 | 20.21±6.00 |
| ALP, U/L | 73.96±15.19 | 42.89±11.37 |
| γ-GT，U/L | 61.32±13.51 | 30.18±5.71 |
| TG, U/L | 2.25±0.43 | 1.07±0.34 |
| TC, U/L | 5.14±0.60 | 3.30±0.34 |
| Platelet, 10^9^/L | 205.78±46.23 | 254.75±37.04 |
| FIB-4 | 2.48±0.59 | 1.08±0.31 |
| **Histological characteristics** |  |  |
| NAS (mean±SD) | 5.68±1.21 | 0 |
| Steatosis grade 1/2/3 | 13/9/18 |  |
| Lobular inflammation 0/1/2/3 | 10/13/10/7 |  |
| Ballooning 0/1/2 | 6/21/13 |  |
| Fibrosis stage 0/1/2/3/4 | 6/10/8/12/4 |  |

BMI, Body Mass Index; ALT, Alanine aminotransferase; AST, Aspartate aminotransferase; ALP, Alkaline phosphatase; γ-GT, γ-glutamyl-transferase; TG, Triglyceride; TC, Total cholesterol; FIB-4, Fibrosis-4 score; NAS, NAFLD Activity Score.

**Table S2. Primers used in this study (H, denotes human and M, denotes mice).**

| **Primer sequences** | | |
| --- | --- | --- |
| **Gene Symbol** | **Forward Primer** | **Reverse Primer** |
| *H-IRF7* | CCCAGCAGGTAGCATTCCC | GCAGCAGTTCCTCCGTGTAG |
| *H-TNFA* | GAGGCCAAGCCCTGGTATG | CGGGCCGATTGATCTCAGC |
| *H-IL6* | CCTGAACCTTCCAAAGATGGC | TTCACCAGGCAAGTCTCCTCA |
| *H-β-ACTIN* | CATGTACGTTGCTATCCAGGC | CTCCTTAATGTCACGCACGAT |
| *M-Irf7* | GAGACTGGCTATTGGGGGAG | GACCGAAATGCTTCCAGGG |
| *M-Tnfa* | CCCTCACACTCAGATCATCTTCT | GCTACGACGTGGGCTACAG |
| *M-Il6* | TAGTCCTTCCTACCCCAATTTCC | TTGGTCCTTAGCCACTCCTTC |
| *M-Ifna1* | GGATGTGACCTTCCTCAGACTC | ACCTTCTCCTGCGGGAATCCAA |
| *M-Ifnb1* | CAGCTCCAAGAAAGGACGAAC | GGCAGTGTAACTCTTCTGCAT |
| *M-Acta2* | GTCCCAGACATCAGGGAGTAA | TCGGATACTTCAGCGTCAGGA |
| *M-Col1a1* | GCTCCTCTTAGGGGCCACT | CCACGTCTCACCATTGGGG |
| *M-Timp1* | CGAGACCACCTTATACCAGCG | ATGACTGGGGTGTAGGCGTA |
| *M-Tgfb1* | CTCCCGTGGCTTCTAGTGC | GCCTTAGTTTGGACAGGATCTG |
| *M-β-Actin* | GTGACGTTGACATCCGTAAAGA | GCCGGACTCATCGTACTCC |

**Supplementary Figure 1 (Related to Fig. 1). The severity of liver disease in patients with MASH was positively correlated with IRF7 expression.**

**a** Representative immunohistochemistry images of H&E, Oil red O staining, and Sirius red staining in liver tissues from NASH patients and normal controls. Scale bar = 50μm, n = 6/group. **b** Graphs show the correlation between the mRNA levels of *IRF7* and NAFLD activity score and Fibrosis score in patients with NASH (n = 40).

**Supplementary Figure 2 (Related to Fig. 3). The metabolic characterisation of the macrophage-specific *Irf7* knockout mice.**

**a** Serial body weight measurement over 16 weeks feeding period (n = 6 mice/group). **b** Quantification of average daily caloric intake over 16 weeks HFHCD feeding period (n = 6 mice/group). **c**-**d** Blood glucose curves during glucose tolerance test (GTT) and corresponding GTT-AUC. **e**-**f** Blood glucose profiles during insulin tolerance test (ITT) and corresponding ITT-AUC. AUC was calculated using the trapezoidal rule. n = 6/group.

**Supplementary Figure 3 (Related to Fig. 5). Macrophage IRF7 induces pro-inflammatory cytokine expression in hepatocytes.**

**a** *Tnfa* mRNA in primary WT hepatocytes co-cultured with *Irf7^ΔMΦ^* and *Irf7^FL/FL^* macrophages under PAOA medium.

**Supplementary Figure 4 (Related to Fig. 8). The knockdown efficiency in different liver cell types after *Irf7* siRNA administration.**

**a** The RNA knockdown efficiency in different liver cell types isolated from liver tissues of WT mice with MCD-induced MASH injected with NS-siRNA or *Irf7*-siRNA (n = 6 mice/group). **b** The protein levels of IRF7 in different liver cell types isolated from liver tissues of WT mice with MCD-induced MASH injected with NS-siRNA or *Irf7*-siRNA (n = 6 mice/group). Data are presented as means ± SDs, and statistical analysis was performed using ANOVA; **P* < 0.05.

**Supplementary Figure 5 (Related to Fig. 8). The metabolic characterisation of the macrophage-specific *Irf7* knockdown mice.**

**a** Serial body weight measurement over 16 weeks feeding period (n = 6 mice/group). **b** Quantification of average daily caloric intake over 16 weeks HFHCD feeding period (n = 6 mice/group). **c**-**d** Blood glucose curves during glucose tolerance test (GTT) and corresponding GTT-AUC. **e**-**f** Blood glucose profiles during insulin tolerance test (ITT) and corresponding ITT-AUC. AUC was calculated using the trapezoidal rule. n = 6/group.

**Supplementary Figure 6 (Related to Fig. 8). Pharmacological intervention with GLP-1 receptor agonist could suppress IRF7 expression and alleviate MASH progression.**

**a** Saline or liraglutide was injected via tail vein. WT male mice fed either an MCD diet to induce experimental MASH were injected with liraglutide, and their livers were subsequently collected for H&E staining, Oil Red O staining, and α-SMA immunohistochemical analysis (scale bar = 50 µm). **b** NAS, serum ALT, and hepatic TG were measured in MCD diet-fed mice treated with saline or liraglutide, and the α-SMA-positive areas were quantified (n = 6 mice/group). **c** qRT-PCR was performed to assess the expression levels of *Acta2*, *Col1a1*, and *Timp1* in liver tissues from WT mice with MCD-induced MASH injected with saline or liraglutide (n = 6 mice/group). **d** qRT-PCR was performed to assess the expression levels of *Acta2*, *Col1a1*, *Timp1* and *Irf7* in liver tissues from WT mice with MCD-induced MASH injected with saline or liraglutide (n = 6 mice/group). Data are presented as means ± SDs, and statistical analysis was performed using ANOVA; **P* < 0.05.

**Graphical Abstract**

Our study identified the reduction of IRF7 in macrophage as an innovative therapeutic target for MASH. Experiments in vivo indicated that IRF7 significantly expressed in the liver tissues of MASH patients and that macrophage-specific *Irf7* knockout attenuated MASH in mice. Furthermore, subsequent experiments demonstrated that *Irf7* knockout suppressed macrophage type I IFN-dependent immune responses. In addition, IRF7 targeted the promoter of inflammatory genes (*Tnfa* and *Il1b*) and promoted proinflammatory gene transcription. Moreover, IRF7 bound to SMAD3 and targeted the *Tgfb1* promoter, thus promoting *Tgfb1* transcription. Ultimately, IRF7 enhanced the secretion of proinflammatory and profibrotic cytokines, leading to hepatic steatosis and activation of HSC. Additionally, the ubiquitin ligase E3 HUWE1 bound to IRF7, increasing IRF7 monoubiquitination and entry into the nucleus to promote downstream transcription. IRF7 disruption blocked MASH progression. Above findings highlight the potential of targeting IRF7 or its regulatory pathways to develop effective therapies.
